# Magnetic Nanostructures for the Removal of Emerging Organic and Inorganic Pollutants: An Overview of Applications in Contaminated Water

**DOI:** 10.3390/ma19061057

**Published:** 2026-03-10

**Authors:** Raquel Murillo-Ortíz, María J. Martínez-Carreón, Rosario Herrera-Rivera, Deyani Nocedo-Mena, Eduardo G. Pérez-Tijerina

**Affiliations:** Centro de Investigación en Ciencia Fisico Matematicas, Universidad Autonoma de Nuevo Leon, Pedro de Alba S/N, Ciudad Universitaria, San Nicolas de los Garza C.P. 66455, Mexico; maria.herrerarv@uanl.edu.mx (R.H.-R.); deyani.nocedomn@uanl.edu.mx (D.N.-M.); eduardo.pereztj@uanl.edu.mx (E.G.P.-T.)

**Keywords:** environmental remediation, magnetic nanostructures, water treatment

## Abstract

Magnetic nanomaterials (MNMs) have been adopted as effective platforms for water remediation owing to their excellent surface-area-to-volume ratios, tunable surface chemistry, and magnetic separability. This review highlights the recent progress made in the synthesis, properties, and environmental applications in the removal of organic and inorganic contaminants using magnetic nanoparticles (MNPs) and one-dimensional magnetic nanofibers. Demonstrated removal rates of organic contaminants such as dyes, pharmaceuticals, and pesticides are often up to 85–100% under laboratory conditions, with adsorption capacities of 580 mg·g^−1^ for melanoidin, 397.43 mg·g^−1^ for Congo Red, and 392.64 mg·g^−1^ for tetracycline. For heavy metals such as As(V), Cd(II), Cr(VI) and Pb(II), efficiencies are generally between 90–99% with maximum adsorption capacities of 909.1 mg·g^−1^ for Pb(II). In particular, the review compares major synthesis routes such as coprecipitation, hydrothermal, solvothermal, thermal decomposition, sol–gel, microwave, and green methods by evaluating their effect on particle size (6–50 nm), magnetic properties (saturation magnetization up to ~101 emu·g^−1^), and removal performance. The four principal mechanisms are described in this paper—adsorption, filtration, transformation, and photocatalysis—giving special emphasis to the advantages of magnetic recovery and advanced oxidation processes. Although most studies remain at the laboratory scale, MNMs demonstrate strong potential for scalable wastewater treatment, provided that toxicity, life-cycle impacts, and matrix effects are carefully evaluated.

## 1. Introduction

Considering the growing global population, the availability of freshwater is expected to decrease over time, which may negatively impact socioeconomic development in many countries. Recent assessments indicate that water scarcity and poor water quality are related to lower agricultural yields, escalating public health threats, and economic fragility in water-stressed regions; thus, sustainable water management has become imperative [1]. Therefore, wastewater reuse has emerged as a viable strategy for ensuring a safe and sustainable water supply.

Magnetic nanomaterials are a versatile class of materials with tunable functional properties and applications in diverse fields. They have been studied for the removal of water contaminants in recent years [2], specifically organic pollutants (e.g., pesticides, dyes, pharmaceuticals) and inorganic contaminants, especially heavy metals (e.g., arsenic, cadmium, lead, and mercury). Other conventional wastewater treatment methods like coagulation–flocculation, membrane filtration, and biological processes all have drawbacks such as high operational costs, sludge production, incomplete removal of emerging contaminants, and low selectivity for trace pollutants, which have driven the development of novel nanomaterial-based technologies [2].

Nanomaterials (NMs) with sizes ranging from 1–100 nm have unique physicochemical properties with respect to bulk materials, which enable more effective contaminant and toxic element removal than conventional treatment. In particular, MNMs characterized by customizable surface and magnetic behavior (ferromagnetic, paramagnetic, or superparamagnetic), have been widely applied in various water treatment applications [3]. Recent studies have focused on the use of various magnetic nanostructures for the removal of inorganic and organic pollutants, including evaluations of removal efficiencies, adsorption capacities, and underlying mechanisms involved in contaminant separation and degradation in wastewater systems [4]. Their magnetic characteristics, such as domain structure, hysteresis, and superparamagnetism, allow for effective separation and recovery by external magnetic fields in order to reduce secondary waste generation and enhance reusability [4].

This review outlines the scope of magnetic nanostructures, including nanoparticles (NPs) and one-dimensional magnetic nanofibers, for the removal of emerging organic and inorganic pollutants from contaminated water through adsorption, photocatalysis, and catalysis. It covers synthesis methods, removal strategies, adsorption mechanisms, and performance evaluations such as efficiencies and capacities. The paper is organized as follows: Section 2 reviews the magnetic properties of nanomaterials; Section 3 discusses their environmental applications; subsequent sections detail synthesis approaches, specific pollutant removal mechanisms, challenges, and future perspectives.

Therefore, this review aims to summarize current synthesis approaches for magnetic nanostructures and identify the most effective mechanisms for contaminant degradation and removal based on recent literature.

## 2. Magnetic Properties of Nanomaterials

The magnetic behavior of NMs is primarily governed by their response to an applied magnetic field, which serves as a fundamental criterion for distinguishing different magnetic regimes. At the nanoscale, the orientation and interaction of magnetic moments within individual particles play a crucial role in determining the overall magnetic character of the material, allowing for the identification of distinct forms of magnetism [5]. The macroscopic magnetic response of these systems can be described by the relationship between magnetic induction (B) and the applied magnetic field (H) [6]. In many materials, this relationship is approximately linear [7], reflecting the intrinsic magnetic permeability (μ), which is commonly expressed as:

(1) B=μH

Materials exhibit different magnetic responses depending on the value of μ. Systems with μ > 1 display paramagnetic behavior, whereas those with μ < 1 are classified as diamagnetic; in free space, μ equals unity. Magnetic properties are often more conveniently described using magnetic susceptibility, χ, which represents the deviation of permeability from unity, where $\mu_{r}$ denotes the relative permeability:

(2) $$\chi =\mu_{r}-1$$

Within this framework, paramagnetic NPs are characterized by positive susceptibility values, while diamagnetic materials exhibit negative susceptibility and vacuum conditions correspond to χ=0. A defining characteristic of MNPs is their tendency to exhibit superparamagnetism at sufficiently small dimensions. This phenomenon is particularly advantageous as it enables strong magnetic responsiveness under an external field while eliminating residual magnetization once the field is removed. As a result, superparamagnetic NPs demonstrate enhanced colloidal stability and dispersion due to the absence of interparticle magnetic attraction. When particle sizes are reduced to approximately 15 nm or below, long-range cooperative ferromagnetic ordering is suppressed, and permanent magnetization is no longer retained after removal of the applied field. Despite this loss of permanent magnetization, such NPs continue to display pronounced magnetic responsiveness with high susceptibility values [8].

**Figure 1:**
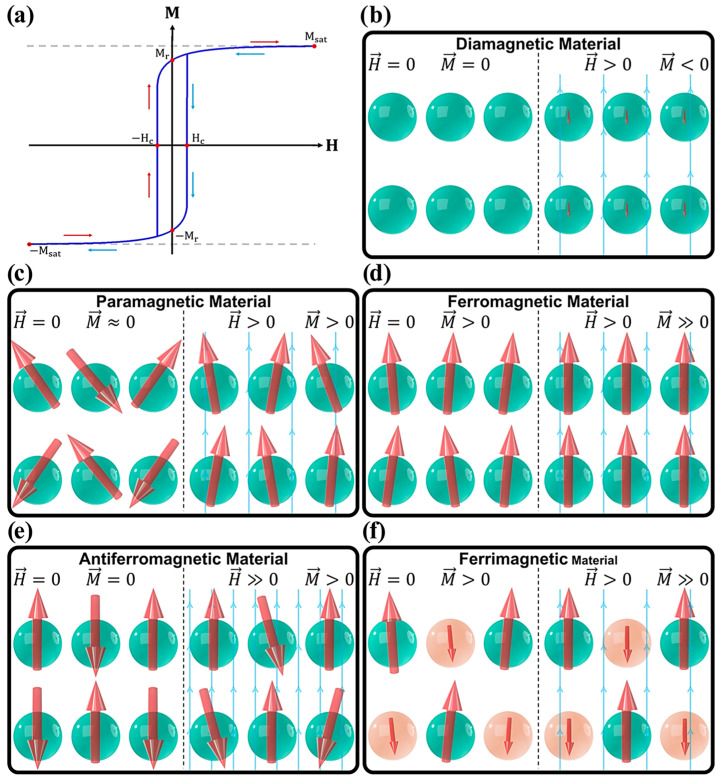
(**a**) Principal magnetic parameters extracted from an M(H) hysteresis loop. (**b**–**f**) Classification of magnetic materials illustrating the alignment of magnetic moments in the absence and presence of an external magnetic field H. Reprinted from Reference [9] under a Creative Commons (CC BY) license.

Ferromagnetism arises from the collective alignment of unpaired electron spins and cannot occur in isolated atoms. Instead, it emerges only when a sufficient number of atoms interact strongly to establish long-range magnetic order. Consequently, when the dimensions of ferromagnetic materials are reduced below a critical size threshold, this collective behavior is disrupted, leading to a transition from ferromagnetic to superparamagnetic behavior. These size-dependent magnetic responses and their associated spin configurations are illustrated in Figure 1.

## 3. Environmental Applications of Magnetic Nanomaterials

**Figure 2:**
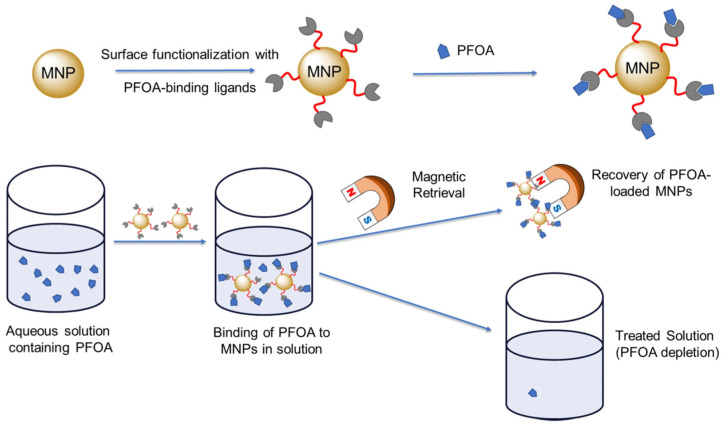
Schematic representation of the functionalization of MNPs with surface ligands for Perfluorooctanoic Acid (PFOA) binding and their magnetic retrieval process following PFOA removal. Reprinted from Ref. [10] under a CC BY license.

Magnetic nanomaterials have been widely applied in environmental remediation and have demonstrated favorable performance in pollution removal and toxicity reduction. Among the most commonly used MNPs are materials with diverse compositions and surface functionalizations that enable adaptation to specific environmental challenges. These materials include pure metals like cobalt, nickel, and iron, metal oxides such as Fe_3_O_4_ and γ-Fe_2_O_3_, and ferrites with the formula MFe_2_O_4_ (where M = Cu, Mg, Mn, Ni). For instance, Figure 2 illustrates how surface functionalization with various ligands enhances interactions between magnetic nanomaterials and target contaminants.

MNMs are widely employed in solid-phase magnetic extraction, an analytical technique used to separate and preconcentrate organic and inorganic analytes from solution. In this process, magnetic adsorbents are introduced into the sample and allowed to interact with the target analytes for a defined period to facilitate adsorption. Subsequently, the adsorbents are removed from the solution using an external magnetic field [11]. The retained analytes can then be recovered from the particle surface by elution using suitable solvents or separation agents. Owing to these advantages, MNMs have attracted significant interest in environmental research applications.

Although MNMs offer significant advantages for water remediation, particularly magnetic separability and high surface reactivity, their potential toxicity and environmental fate after application must be carefully considered. The increasing pressure on freshwater resources due to water scarcity and associated socioeconomic impacts highlights the need for sustainable and safe treatment technologies [1]. Although MNMs have been identified as effective tools for pollutant removal and rapid magnetic recovery, several challenges remain, including nanoparticle persistence, surface transformation, aggregation, and metal ion leaching once released into aquatic environments [2].

Previous studies have confirmed that nanoparticle toxicity is strongly dependent on size, surface chemistry, and composition, and that oxidative stress and ion dissolution are key mechanisms of ecotoxicity [3,11]. Magnetic parameters governing controlled separation, such as domain structure, hysteresis characteristics, and superparamagnetism, can significantly reduce environmental dispersion when appropriate recovery strategies are implemented [4,7]. Moreover, particle interactions at fluid interfaces and within complex matrices influence aggregation, sedimentation, and long-term stability, thereby affecting environmental mobility [5]. Ferrite-based materials and surface-functionalized coatings have been proposed to enhance stability and minimize undesirable transformations [6,9]. More recently, magnetically retrievable nanoparticles coupled with specific ligands have improved selectivity and recovery from natural water matrices, thereby reducing the risk of secondary contamination [10].

However, comprehensive life-cycle evaluations and standardized ecotoxicological assessments remain critical components for the safe adoption of MNMs in large-scale environmental applications [2,11].

## 4. Synthesis and Processing of Magnetic Nanoparticles

Substantial advances have been achieved in the synthesis of MNPs, driven by the need to precisely control parameters such as particle size, shape, morphology, surface characteristics, chemical composition, stability, and biocompatibility. On this basis, synthesis strategies are generally grouped into two primary categories: chemical and physical methods. Physical methods typically enable the formation of smaller particles with more uniform size distributions, whereas chemical methods tend to produce NPs with relatively larger dimensions and broader size distributions [12]. Each of these classifications is described below along with their respective techniques.

### 4.1. Physical Methods

#### 4.1.1. Ball Milling Method

This method is commonly used for the production of polycrystalline samples. It consists of mixing powders and using the mechanical energy of the mill to create alloys, induce solid state chemical reactions, or reduce particle size.

The process is carried out by placing a mixture of elemental or pre-alloyed powders together with one or more balls (the material can be high-hardness steel, stainless steel, or ceramic) in a container made of agate to prevent contamination from grinding, and finally the NPs are obtained by sintering at high temperatures [13].

Bedoya et al. synthesized Fe_3_O_4_ NPs using the ball-milling technique. The nanoparticles obtained had sizes ranging from 6 to 12 nm, with a high saturation magnetization of 55.57 emu/g [14].

#### 4.1.2. Laser Ablation Method

The laser ablation method is a fast and versatile technique that uses a laser (an acronym for Light Amplification by Stimulated Emission of Radiation) as the energy source for the ablation of solid materials. In this process, coarse particles in the micrometer or nanometer size range are used as raw materials and are evaporated under a focused laser beam. Extremely high energy is concentrated at a localized point on the solid surface, leading to evaporation of the light-absorbing material. Ablation describes the process by which atoms are removed from a surface and involves not only a single-photon process (breaking chemical bonds) but also multiphoton excitation (thermal evaporation). This approach also allows for the simultaneous analysis of a wide range of concentrations [15]. Piotto and collaborators reported the synthesis of M-type SrFe_12_O_19_ MNPs with nanoparticle sizes of 80 nm [16].

#### 4.1.3. Electrical Wire Explosion Method

It is a highly productive method (up to 200 g) that yields powders with an average particle size ranging from 20 to 100 nm and requires an energy consumption of approximately 25 kWh/kg. The process begins when a high-density (10^4^–10^6^ A/mm^2^) current pulse, usually produced by the discharge of a capacitor bank, reaches the lower contact plate. The voltage source discharges, thereby evaporating the portion of the wire located between the two electrodes. After each explosion, the feeding device advances the wire to repeat the process and thus produces the nanoparticles [17]. Song et al. reported the synthesis of iron oxide NPs using the wire explosion technique [18].

#### 4.1.4. Electron Beam Lithography Method

Instead of using a light beam to create the desired pattern on a surface coated with an electron-sensitive resist, electron beam lithography employs a focused electron beam. High-energy electrons can fabricate extremely fine features at the nanometer scale because the electron wavelength is much shorter than that of visible light, and the beam diameter can be on the order of a few nanometers. It is a flexible and relatively cost-effective method that can create NPs with distinct shapes [19]. This method has been widely used to produce magnetic nanorings and nanorods from thin metallic films spin-coated with an organic resin [20].

### 4.2. Chemical Methods

#### 4.2.1. Precipitation and Coprecipitation Method

This is a conventional and well-established method for the synthesis of metal oxide NPs, which is carried out using solutions of precursor salts, typically chlorides and nitrates, in aqueous or acidic media. Coprecipitation of metal ions is achieved by adding alkaline aqueous solutions like ammonium hydroxide or sodium hydroxide. The resulting precipitate is then washed and filtered to remove residual salts and hydroxides formed during the reaction, and in some cases, it is subsequently calcined to obtain the oxide of interest [21]. Recently, α-Fe_2_O_3_ NPs with nanoparticle sizes of 28.87 nm were obtained by coprecipitation [22].

#### 4.2.2. Sol–Gel Method

The Sol–Gel method is an economical and versatile route for the synthesis of MNPs. The molecular precursor, typically a metal alkoxide, is dissolved in alcohol or water and converted into a gel through alcoholysis and hydrolysis under heating and stirring. In general, it can be described in five key steps: hydrolysis, polycondensation, aging, drying, and thermal decomposition. The method can be carried out at low temperatures and produces materials with high purity, good crystallinity, and homogeneity [23]. Recently, Thair and collaborators synthesized MNPs using the Sol–Gel method with average nanoparticle sizes of 29 nm and magnetic properties M_s_ = 1.72 emu/g and coercivity H_c_ = 1040 G [24].

#### 4.2.3. Hydrothermal Method

The hydrothermal method involves heterogeneous reactions in organic solvents or mineralizers under high-pressure and high-temperature conditions. This enables the dissolution and subsequent recrystallization of materials that would not normally dissolve. This technique is widely used for producing high-purity NMs because it is relatively simple. A high degree of control can be achieved, allowing synthesis parameters to be adjusted to tailor the shape and size of the resulting materials [25]. The process is flexible and readily adjustable. For example, da Silva [26] reported the synthesis of spherical Fe_2_O_3_ NPs with a mean diameter below 10 nm.

#### 4.2.4. Solvothermal Method

Solvothermal synthesis is analogous to the hydrothermal method; however, it employs an organic solvent instead of an aqueous medium. This substitution significantly expands the range of materials and enables reactions typically associated with the hydrothermal method. Several parameters are critical, including solvent selection, precursor choice, reaction temperature, reaction time, and aging duration. These factors collectively determine particle size, morphology, and distribution. An important advantage of solvothermal synthesis is its ability to minimize the adsorption of anions and cations on the surfaces of nanoparticles, thereby reducing contamination commonly observed in other methods [27]. Sahadevan et al. reported the synthesis of Fe_3_O_4_ and Fe_2_O_3_ NPs with particles averaging 15.16 nm [28].

#### 4.2.5. Microemulsion Method

This method is used for the synthesis of nanoparticles based on microemulsions, which are thermodynamically stable isotropic dispersions of two immiscible liquids, water and oil, stabilized by a monolayer film of surfactant molecules surrounding each microdomain [29]. Three types of microemulsions can be distinguished: (1) oil-in-water (o/w), with water as the continuous phase and dispersed oil droplets; (2) water-in-oil (w/o), with oil as the continuous phase and dispersed water droplets; and (3) systems containing comparable amounts of oil and water. Magnetic materials produced by the microemulsion method are typically obtained in limited quantities and exhibit uniform dispersion. Bozcan synthesized α-Fe_2_O_3_ NPs with an average particle size of 13.1 nm and reported that the method is simple and effective [30].

#### 4.2.6. Polyol Method

The polyol method is a widely used technique that involves the use of an inorganic metal compound or salt, which is dispersed in a polyol or polyalcohol solvent. The suspension is stirred and heated to specific temperatures until the particles precipitate. The solid precursor, which can be highly soluble (nitrate, chloride, acetate) or only slightly soluble (oxide, hydroxide), is suspended in the liquid polyol. The type of precursor and the concentration of the reducing agent significantly influence nucleation, growth, agglomeration, as well as particle size distribution. This method allows control over the nucleation and growth stages during synthesis and enables the production of particles with defined characteristics, uniform shape, nanometric size, narrow size distribution, and a low degree of agglomeration. However, this process is slow and not readily applicable on a large scale [31]. Anit et al. synthesized magnetic hematite nanoparticles in different phases, α and γ phases, with a mean particle diameter of 41 nm. The authors concluded that this method can be adapted to add value to waste products in industry [32].

#### 4.2.7. Thermal Decomposition Method

This method is based on the synthesis of nanoparticles through the transformation of precursors with high melting temperatures. Typically, thermal decomposition reactions are carried out at elevated temperatures ranging from 200 to 320 °C in high boiling organic solvents under an inert atmosphere. The thermal decomposition of organometallic precursors is conducted in the presence of organic surfactants. Consequently, the resulting materials display high crystallinity, controlled particle size, and well-defined morphology [33]. Although this method enables excellent control over crystallinity and morphology, the high reaction temperatures and prolonged heating times increase energy consumption compared with hydrothermal synthesis, which is generally performed at lower temperatures (120–220 °C) in aqueous media under autogenous pressure. Agarwal et al. reported the preparation of Fe_2_O_3_ MNPs in oleic acid and proposed a hematite nanooctahedron growth mechanism after 24 h of reaction [34].

#### 4.2.8. Spray Pyrolysis Method

This method involves a liquid precursor solution that is dispersed by a gas stream to form an aerosol, which evaporates upon passing through a flame at temperatures above 1200 °C. The resulting vapor phase reacts to form primary particles, which subsequently undergo growth until they are collected as nanoparticle aggregates. Operational parameters such as feed flow rate, dispersion conditions, and precursor concentrations determine the properties of the nanoparticles [35]. Previous studies have reported the synthesis of maghemite (γ-Fe_2_O_3_) nanostructures with particle sizes ranging from 6 to 41 nm [36].

#### 4.2.9. Pechini Method

In this method, α-hydroxycarboxylic acids such as citric acid, form chelates with metal cations, which subsequently participate in polymerization with a polyhydroxy alcohol. The principle of the Pechini method is to obtain a precursor polymer resin containing branched macromolecules within which the cations are uniformly distributed. Heating the resin to 300 °C causes it to decompose, thereby forming the corresponding metal oxide. In this process, a dehydration reaction occurs between a dicarboxylic acid and a diol [37]. Murillo-Ortiz and colleagues reported the synthesis of SrFe_12_O_19_ MNPs using the Pechini method, with a mean particle diameter of 78 nm [38].

#### 4.2.10. Self-Assembly Method

The self-assembly method is a simple technique in which oppositely charged polyelectrolytes are alternately adsorbed onto core materials. The films are assembled by repeated layer-by-layer adsorption of oppositely charged functional components. The technology exploits electrostatic interactions between the substrate and polyelectrolyte monolayers to create multilayer nanostructures held together by electrostatic forces. The formation of these systems is attributed to electrostatic and hydrophobic interactions, hydrogen bonding, and van der Waals forces [39]. This method enables the formation of multilayer molecular structures, the formation of nanoparticles smaller than 20 nm, and precise control of stoichiometry [40].

#### 4.2.11. Laser Pyrolysis Method

This technique is based on heating organometallic vapors using a laser beam. The reactant vapors rapidly decompose, releasing atoms that form clusters due to collisions with inert gas molecules. This method produces very small particles with varying degrees of crystallinity, spherical shape, and homogeneous size distribution. However, due to rapid nucleation and the immediate transport of particles by carrier gases out of the reaction channel, the nanoparticles do not have sufficient time to undergo growth or a crystallization stage. As a result of the short residence time in the reaction channel, NPs prepared by laser pyrolysis are typically smaller and less crystalline than those obtained by previously described methods [41]. Crivenanu reported the synthesis of iron oxide NPs with particle diameter below 4 nanometers and a high saturation magnetization of 101.4 emu/g [42].

#### 4.2.12. Oxidation-Reduction Method

The oxidation–reduction method produces nanoparticles through the loss of electrons from one atom and their gain by another. The species that supplies electrons is the reducing agent, whereas the species that accepts them is the oxidizing agent. Redox reactions are analogous to proton transfer reactions; however, instead of transferring a proton, electrons are transferred from the reducing agent to the oxidizing agent. In this approach, water may serve as a solvent and act as either an oxidizing agent by being reduced to H_2_ or a reducing agent by being oxidized to O_2_, leading to nanoparticle formation [43]. Oskaya and colleagues reported the synthesis of iron oxide NPs with average particle sizes of 40–50 nm [44].

#### 4.2.13. Microwave Method

The microwave irradiation technique produces nanoparticles with narrow size distribution. Microwave irradiation generates high-frequency electromagnetic fields, which are capable of heating materials containing polar molecules in a solvent or conductive ions in a solid. Polar solvents are heated as their molecular dipoles align with the oscillating field and dissipate energy through molecular collisions [45]. In a study by Nashaat et al., the authors reported the synthesis of M-type SrFe_12_O_19_ NPs at 1000 °C, exhibiting a saturation magnetization of 70 emu∙g^−1^ and a coercive field of 6.3 kOe [46].

#### 4.2.14. Arc Discharge Method

This method enables the synthesis of carbon-encapsulated MNPs or metal-carbide MNPs. In this process, the metal precursor is placed in a cavity within a graphite electrode and evaporated by arc discharge. Owing to limitations such as low efficiency and limited control over particle size and shell thickness, this method cannot be used on an industrial scale [47]. Karami reported the synthesis of magnetite/hematite nanocomposites with particle sizes ranging from 1 to 13 nm [48].

#### 4.2.15. Sonochemical Method

This technique represents a novel approach for the synthesis of nanostructures. In acoustic cavitation, the collapse of gas bubbles generates localized high temperatures and pressures in the solution. The process must meet certain sonochemical conditions (1000–1500 Hz acoustic frequency, 10 cm–100 μm acoustic wavelength). The typical operating frequency ranges from 20 kHz to 15 MHz. In addition, the synthesis process can introduce structural defects within the particles, and the distribution of these imperfections can alter their susceptibility to magnetic fields [49]. In a study by Yadav et al., iron oxide NPs were synthesized at 70 °C for 1 h using an ultrasonicator, with an average particle size of 38.9 nm [50].

#### 4.2.16. Combustion Method

Combustion synthesis in solution is a versatile, simple, and rapid process that allows for the effective synthesis of a variety of nanoscale materials. This method involves a self-sustaining reaction taking place in a homogeneous solution containing oxidants and fuels [51]. One study reported the synthesis of magnetic iron oxide NPs using the combustion method. The authors reported medium-sized particles of 10.7 ± 3.09 nm and a 41.8 emu/g value for saturation magnetization [52].

#### 4.2.17. Chemical Vapor Deposition

This method involves the decomposition of a precursor vapor or gas containing carbon atoms (hydrocarbons) in the presence of a metal catalyst on a substrate. It is a cost-effective method for industrial-scale production, allows for precise control of diameter, and enables the production of vertically aligned magnetic nanostructures [53]. According to Levish et al., nanocrystalline iron oxide NPs were synthesized, which exhibited a high surface-to-volume ratio and enabled simple magnetic separation. The authors concluded that this method can be applied to a wide range of substrates [54].

## 5. Mechanisms of Contaminant Removal by Magnetic Nanomaterials

Currently, several types of MNMs are prepared for environmental treatment, which exhibit varying sizes, morphologies, and chemical properties. These nanoparticles perform four principal degradation/removal processes: adsorption, filtration, transformation, and photocatalysis [55]. Their high specific surface areas and tunable surface chemistries enable these functions. Furthermore, it has been postulated that the pore dimensions and surface structure of these materials are conducive to pollutant adsorption. NMs have superior magnetic characteristics and can facilitate the separation of contaminants from water and promote regeneration, resulting in increased reuse.

Filtration mechanisms involve the physical retention of contaminants through size exclusion, surface trapping, or incorporation of MNMs into porous matrices and membrane systems. Magnetic nanomaterials embedded in polymeric or inorganic supports enhance contaminant capture while allowing easy magnetic recovery, thereby improving operational stability and reuse [55].

Transformation processes refer to the chemical conversion of pollutants into less toxic or more stable forms. For instance, nano zero-valent iron can reduce toxic Cr(VI) to the less harmful Cr(III), combining adsorption and redox mechanisms [55]. Additionally, advanced oxidation processes generate highly reactive species like hydroxyl radicals (^-^OH) and superoxide radicals (O_2_^−^), which oxidize organic contaminants into smaller, less hazardous molecules [56].

Photocatalysis technology enables the photodegradation of inorganic and organic pollutants through an active, lightweight magnetic nanomaterial catalyst that facilitates the degradation of diverse contaminants in water. In a photocatalytic system with a semiconductor material as the catalyst medium, electron–hole pairs (e^−^-h^+^) are generated upon absorption of light with energy greater than its band gap. These e^−^–h^+^ pairs generate reducing species and radicals or other highly reactive oxidizing species in wastewater (OH^−^ and O_2_^−^), leading to the degradation of organic matter and inorganic pollutants in wastewater [56]. These magnetic nanophotocatalysts have a higher surface-area-to-volume ratio than conventional photocatalysts, ensuring that photogenerated electrons and holes are readily available at the surface. Consequently, nanostructured semiconductor materials are more effective for treating contaminated water than conventional ones [57].

## 6. Strategies for Removing Contaminants

The benefits of applying MNMs have led to major advances in environmental remediation. Researchers have proposed using various approaches to solve water treatment problems, such as nanomaterial-based strategies involving MNPs and polymeric nanofibers. The following sections describe each of these strategies.

MNPs are of significant research interest due to their diverse properties. Their nanoscale size and high surface-area-to-volume ratio enable high removal capacity, fast kinetics, and strong reactivity toward contaminants. The magnetic properties offer the potential for a more cost-effective and convenient separation process using an external magnetic field compared with conventional separation methods used to recover such small particles. A summary of the strengths and weaknesses of each synthesis method for the different types of MNPs is included in Table 1.

## 7. Classification of Emerging Pollutants

Recently, the presence of emerging pollutants in the environment has increased rapidly; consequently, their impact on human and ecological health has become considerable, making the implementation of appropriate detection and removal techniques essential [80,81]. The following subsections describe the two major classifications of emerging pollutants: organic and inorganic.

### 7.1. Organic Pollutants

Organic contaminants present in drinking water pose a serious risk to human health, including endocrine disruption, carcinogenicity, and bioaccumulation. Industrial effluents containing diverse organic contaminants, such as pesticides, dyes, nitrogenous compounds, and phenolics—frequently detected in polluted waters—exacerbate this issue [82]. In addition, pollutants (pharmaceuticals, personal care products, perfluoroalkyl acids) persist and resist removal by existing wastewater management technologies, often resulting in removal efficiencies below 50%, further compromising aquatic habitats and public health.

Consequently, to enhance environmental remediation, it is essential to develop efficient and scalable methods. Collectively, these findings underscore the high efficiency and capacity of MNMs, positioning magnetic nanoadsorption as a versatile mechanism for contaminant capture from drinking water and wastewater, complemented by photocatalytic degradation using MNPs to achieve complete mineralization.

Table 2 summarizes the removal of different organic contaminants using MNP-based adsorbents.

### 7.2. Inorganic Contaminants

Inorganic pollutants include salts, metals, and other non-carbon-containing compounds. Metal ions such as mercury(II), lead(II), chromium (III), chromium (VI), nickel(II), cobalt(II), copper(II), cadmium(II), silver(I), arsenic(V), arsenic(III), fluoride (F^-^) and others are ecotoxicologically hazardous. They can be efficiently removed from wastewater through the application of magnetic nanoparticles. Furthermore, these NMs enable easy recovery of the adsorbent material as this approach has proven to be simple and cost-effective [152]. Several studies conducted by different authors have evaluated the capacity of various MNPs to remove inorganic pollutants, and these findings are summarized in Table 3.

Most studies on contaminant removal have been conducted using synthetic solutions or simulated wastewater rather than real wastewater. Additionally, nanoparticles can facilitate the simultaneous removal of multiple contaminants, thereby improving overall treatment efficiency.

## 8. Polymeric Nanofibers with Magnetic Nanoparticles

Currently, significant advances in nanotechnology have drawn considerable attention to one-dimensional (1D) NMs, particularly nanofibers, along with their application and preparation methods. Due to their advantageous properties, such as a high surface area, interconnected structure, high porosity, and functionalization capabilities, they are widely used in various fields of research. In environmental remediation, this technology can be used to develop novel materials for the elimination of harmful pollutants, thereby gradually reducing water scarcity and pollution [188].

The literature reports several techniques for manufacturing polymeric nanofibers, including pattern processing, stencil-assisted synthesis, solvent melting, phase separation, and electrospinning. Each of these fabrication techniques is described below.

### 8.1. Electrospinning Technique

Electrospinning stands out as a highly versatile and efficient technique for fabricating continuous nanofibers and nanostructured materials, offering an exceptionally high surface-to-volume ratio (10–40 m^2^/g), tunable porosity (>80%), and dimensions spanning from nanoscale to microscale. These nanofibers can be fabricated from a wide range of polymeric materials.

This method is particularly powerful due to its precise functionalization capabilities through different surface chemistries, enabling the easy incorporation of MNPs and other functional NMs, thereby markedly increasing adsorption capacity, selectivity, and recyclability for the removal of inorganic and organic contaminants from wastewater. Common components of an electrospinning system include a high-voltage power supply (10–30 kV), an infusion pump for controlled delivery of the polymer solution, a metal-tipped needle as the spinneret, and a grounded aluminum collector plate.

Nanofibers are fabricated by dissolving the chosen polymer in an appropriate solvent to form a viscoelastic solution into which nanoparticles (e.g., magnetic Fe_3_O_4_), peptides or drugs can be incorporated to tailor the material for specific environmental remediation applications [189].

### 8.2. Self-Assembly

This method is a low-throughput, bottom-up technique in nanotechnology in which molecular components (peptides, block copolymers, and surfactants) are designed and prepared to arrange themselves into highly ordered, hierarchical structures, such as nanofibers, under thermodynamically favorable conditions. This organization is facilitated largely by various non-covalent interactions such as electrostatic (ionic) interactions, hydrogen bonding, hydrophobic effects, π–π stacking, weak coordination bonds, and van der Waals forces, enabling molecular-scale control over morphology, diameter (usually under 100 nm), and functionalities.

Self-assembly, in the case of environmental remediation, is particularly effective due to its ability to fabricate thinner, more uniform, and multifunctional nanofibers designed to encapsulate MNPs, such as Fe_3_O_4_. These nanostructures exhibit superparamagnetic properties, enabling easy magnetic separation, enhanced selectivity, and improved recovery and reuse of the adsorbent.

Nevertheless, the key limitations of this approach—low production yields, scalability challenges—and intricate molecular design and synthesis protocols hinder the transition of this method from laboratory to industrial scales [190].

### 8.3. Phase Separation

Phase separation is a thermally induced versatile process for fabricating nanofibers, which is particularly suitable for the development of porous nanofibrous scaffolds based on well-designed microstructures.

The method begins with a homogeneous polymer solution prepared in an appropriate solvent, e.g., tetrahydrofuran. Phase separation is then induced by introducing a non-solvent or by applying heat treatment, resulting in the formation of a polymer-rich phase (usually on top) and a solvent-rich phase (on the bottom), which leads to gelation [191].

The resulting gel is frozen and then freeze-dried (lyophilized) to sublimate the solvent to produce a very porous network of nanofibers. Key parameters such as solvent type, phase separation temperature, polymer concentration, and freezing conditions must be adjusted for effective tuning of fiber diameter, pore size, interconnectivity, and overall morphology.

The minimal equipment requirements of this method, together with its ability to produce nanofibers with high surface area and porosity, sometimes exceeding 90%, enhance their adsorption capacity and make them suitable for environmental remediation, such as recovering heavy metals and organic matter from wastewater.

In addition, MNPs (such as Fe_3_O_4_) can be uniformly dispersed in the polymer solution prior to phase separation, imparting superparamagnetic properties that facilitate magnetic recovery and recyclability, improving selectivity and pollutant removal efficiency while overcoming scalability limitations commonly associated with other techniques, thereby aligning with sustainable water treatment goals.

### 8.4. Template Synthesis

Template synthesis and template-assisted synthesis are highly controlled methods for fabricating uniform one-dimensional nanostructures, such as nanofibers, nanotubes, rods, and wires, with precise control over morphology, diameter (nm to μm), length, and orientation. These approaches can be implemented using either top-down or bottom-up strategies.

These approaches can be used independently or in combination with complementary processes (e.g., chemical vapor deposition, sol–gel chemistry, electrodeposition, and polymerization) for the production of various NMs such as semiconductors, metals, conductive polymers, and carbon nanotubes. It is adaptable to the physicochemical requirements of the target material [192].

The templates are classified into hard and soft types. Hard templates like anodic aluminum oxide membranes, track-etched polycarbonate filters, or colloidal crystals, enable the fabrication of solid rod-like or wire-like nanostructures through precursor infiltration (e.g., polymer solutions containing a high loading of magnetic Fe_3_O_4_ nanoparticles), followed by template removal via selective etching, dissolution, or thermal decomposition. Soft templates, such as surfactants, block copolymers, lipids, or supramolecular assemblies, rely on self-organization processes and can be removed to form hollow nanotubes or complex hierarchical structures.

In environmental remediation, template synthesis plays an important role because it enables the formation of highly ordered and monodisperse nanofibers with tunable surface area, porosity, and functionality. Incorporation of superparamagnetic MNPs facilitates rapid magnetic separation, high adsorption selectivity, and recyclability for the efficient removal of heavy metals, dyes, and organic contaminants from wastewater.

Although template synthesis offers excellent morphological control through adjustable template sizes that enable precise diameter tuning, its scalability remains limited. Structural damage during template removal and lower production throughput compared to electrospinning represent significant challenges.

### 8.5. Drawing

Drawing, also known as wire drawing or glass rod drawing, is a straightforward, low-cost mechanical method for fabricating ultra-long, continuous nanofibers, typically with diameters ranging from 50 nm to several micrometers, making it particularly suitable for producing mechanically robust structures. The process involves placing a polymer droplet (often viscoelastic melts like polyethylene oxide or poly(methyl methacrylate)) on a hydrophilic substrate such as a SiO_2_ surface. A sharp tip, such as a glass rod, micropipette, or tungsten probe, is then dipped into the droplet and slowly withdrawn at controlled speeds (e.g., 0.1–10 mm/s), stretching the polymer into a stable liquid bridge that solidifies into a single, elongated nanofiber upon solvent evaporation or cooling [193]. Recent advancements, including automated single-step systems with motorized stages and imaging feedback, enable continuous high-throughput production of nanofibers exceeding 1 m in length.

In the context of environmental remediation, drawing excels in generating long, uniform magnetic nanofibers by dispersing superparamagnetic NPs (e.g., Fe_3_O_4_) directly into the polymer droplet prior to drawing, imparting magnetic separability, high mechanical strength for durable membranes, and enhanced adsorption performance due to the fibers’ aspect ratio and surface area. The minimal equipment requirements of this method (no high-voltage setup or complex reactors), together with solvent-free variants, align well with sustainable manufacturing principles, aiding in the efficient capture of heavy metals and dyes from wastewater with superior recyclability.

Limitations include challenges in consistently achieving sub-100 nm diameters, sensitivity to polymer rheology and ambient humidity, limited polymer compatibility, and lower scalability compared to electrospinning, although automation alleviates some throughput problems.

### 8.6. Centrifugal Spinning

Centrifugal spinning, also known as centrifugal jet spinning, is a high-throughput, versatile method that leverages centrifugal force—analogous to cotton candy production—to rapidly fabricate diverse nanofibers, including polymeric, ceramic, metallic, and carbon-based variants.

This method often surpasses electrospinning in production speed and yield. The process entails loading a polymer solution or melt into a rotating spinneret reservoir equipped with multiple orifices. Upon high-speed rotation (typically 1000–10,000 rpm), centrifugal force expels the solution as thin jets through the orifices, which stretch, thin, and solidify via rapid solvent evaporation or cooling upon deposition onto a stationary or rotating collector, yielding aligned or random nanofiber mats with diameters tunable from 100 nm to several micrometers through adjusting rotation speed, solution viscosity, orifice size, and collector distance.

Due to the solvent compatibility of this technique, the absence of high-voltage requirements, and scalability, it can be widely used for the industrial synthesis of magnetic nanofiber membranes for environmental remediation [194].

Prior to spinning, magnetic nanoparticles can be uniformly dispersed in the polymer solution, imparting superparamagnetic capabilities that enable easy magnetic separation and recyclability. The resulting nanofibers exhibit high surface area, porosity, and mechanical integrity, which make them effective carriers for the removal of heavy metals, dyes, and organic contaminants from wastewater with removal efficiencies often exceeding 90% in some studies.

Centrifugal spinning provides a 10–100-fold improvement in throughput and demonstrates better suitability for thermoplastics while reducing the need for high-tech equipment and addressing inherent scalability bottlenecks associated with electrospinning. However, challenges remain, including limited precision in fiber placement, uniform collection at ultra-high speeds, difficulty in consistently achieving sub-100-nm diameters, and jet instabilities influenced by ambient humidity. These features make centrifugal spinning a sustainable and economic alternative that meets the demand for large-scale water treatment. A summary of advantages and disadvantages of each nanofibers synthesis method is included in Table 4.

Due to the growing understanding of material processing at the nanoscale, researchers worldwide are proposing the use of magnetic nanostructures in polymer nanofibers because of their novelty and physicochemical properties. These materials are considered valuable for diverse applications, are relatively inexpensive, exhibit excellent magnetic separation selectivity, and offer outstanding performance. Therefore, a summary of some studies recently published in 2022, 2023, 2024, and 2025 is presented in Table 5.

## 9. Isotherms for Equilibrium Sorption Measurements

Adsorption is commonly described by an isothermal equilibrium relationship that correlates the amount of pollutant adsorbed per unit mass of adsorbent at equilibrium (q_e_, mg∙g^−1^) with the equilibrium concentration of the pollutant in solution ($C_{e}$, mg∙L^−1^). An isotherm is considered favorable when its upward convex profile reflects a high adsorption capacity of the solid at low solute concentrations in the liquid phase. Conversely, an isotherm is considered unfavorable when its upward concave profile indicates a low loading on the solid phase throughout the mass transfer zone.

The irreversible isotherm represents a limiting case of highly favorable adsorption behavior, where the sorbed solute concentration remaining in solution becomes negligible. Isotherm models are generally classified into two categories: the first applies to single-component systems such as Freundlich, Langmuir, Sips, and Temkin models, and the second applies to multicomponent systems such as the extended Freundlich model, the extended Langmuir model, and Langmuir–Freundlich combined models [239].

In the context of MNMs, equilibrium modeling is particularly important because nanostructure morphology, surface functionalization, and magnetic interactions directly influence adsorption site distribution and energy heterogeneity. Below is a brief description of the models used in contaminant adsorption.

### 9.1. Freundlich Model

This empirical isotherm model is widely used to describe adsorption on heterogeneous surfaces with nonuniform binding energies, multilayer sorption without a saturation limit, and interactions between adsorbed molecules. It effectively describes adsorption behavior in many nanofiber-based systems for contaminant removal, as shown in Table 5, such as materials like Polyacrylonitrile@carbon/Material of Institut Lavoisier (MIL)-101(Fe) [211]. The model is mathematically expressed as:

(3) $$q_{\;e}=K_{F}{C_{e}}^{1/n}$$

where $K_{F}$ is the Freundlich affinity constant that reflects the adsorption capacity of the adsorbent, and 1/n (dimensionless) represents the adsorption intensity or surface heterogeneity factor.

For instance, magnetic mesoporous carbon/β-cyclodextrin–chitosan nanocomposites used for fluoroquinolone removal exhibited Freundlich behavior, which was attributed to heterogeneous surface energies introduced by polymeric functional groups and magnetic nanoparticle incorporation [240].

### 9.2. Langmuir Model

This model assumes monolayer adsorption onto a surface with a finite number of identical sites, where adsorption occurs uniformly and no interactions exist between adsorbed molecules. Its derivation relies on key assumptions: the maximum adsorption corresponds to complete saturation of the monolayer with the contaminant solute; the adsorption energy is constant across the surface; and there is no transmigration (lateral movement) of solute molecules in the surface plane. Langmuir proposed the following expression, which is widely applicable to nanofiber-based systems for contaminant removal, as shown in Table 5:

(4) $$q_{\;e}=\frac{q_{max}\;bC_{e}}{1+bC_{e}}$$

where q_max_ is the maximum adsorption capacity (mg∙g^−1^), and b is the Langmuir affinity constant reflecting the adsorption energy (L∙mg^−1^).

Functionalized Fe_3_O_4_ nanocomposites for Cd(II) removal have demonstrated Langmuir-type monolayer adsorption behavior due to the relatively uniform distribution of chemically modified adsorption sites [241].

### 9.3. Dubinin-Radushkevich Model

The Dubinin-Radushkevich (D-R) isotherm is an empirical model originally developed to describe adsorption processes following a pore-filling mechanism, which is particularly applicable to both homogeneous and heterogeneous surfaces. Unlike the Freundlich and Langmuir models, it provides insights into the adsorption energy and helps distinguish between physical and chemical adsorption by calculating the mean free energy of adsorption (E), where values of E < 8 kJ/mol indicate physical adsorption and values between 8–16 kJ/mol suggest chemisorption. This model is particularly relevant for nanofiber-based systems for contaminant removal, as demonstrated in Table 5 (e.g., GO/CMC/FeNPs for Pb adsorption with a capacity of 1850 mg/g). The nonlinear form is expressed as Equation (5):

(5) $$q_{\;e}=q_{s}exp\left({-\;K}_{DR}\varepsilon^{2}\right)$$

where $\varepsilon =RTln\left(1+\frac{1}{C_{e}}\right)$ is the Polanyi potential, $q_{s}$ (mg/g) is the theoretical saturation capacity, $K_{DR}$ (mol^2^/J^2^) is the D-R constant related to the mean free energy of adsorption, R is the gas constant (8.314 J/molK), and T is the absolute temperature (K).

The Dubinin-Radushkevich model has been shown to be particularly sensitive to pore-filling mechanisms in magnetic biochar and Fe_3_O_4_-based composites, where magnetization enhances pore accessibility and modifies adsorption energy distributions for inorganic pollutants such as Cr(VI) [242].

### 9.4. Temkin Model

The Temkin isotherm model describes adsorption on heterogeneous surfaces, assuming that the heat of adsorption of all molecules decreases linearly with increasing surface coverage due to adsorbate-adsorbate interactions. Unlike the Langmuir model, which posits uniform adsorption energy across identical sites, the Temkin model accounts for non-uniform bond energies up to a maximum value, making it particularly suitable for heterogeneous adsorbents exhibiting multilayer sorption and molecular interactions. It is frequently expressed in its linearized form as:

(6) $$q_{\;e}=\frac{RT}{b_{T}}ln(K_{T})+\frac{RT}{b_{T}}ln(C_{e})$$

where R is the gas constant (8.314 J/molK), T is the absolute temperature (K), $b_{T}$ is the Temkin isotherm constant related to the heat of adsorption (J/mol), and $K_{T}$ is the Temkin equilibrium binding constant (L/g) corresponding to the maximum binding energy. A linear plot of $q_{\;e}$ versus $ln(C_{e})$ yields a slope of $\frac{RT}{b_{T}}$, indicative of adsorption heat, and an intercept of $\frac{RT}{b_{T}}ln(K_{T})$, which is related to adsorption capacity.

Temkin behavior has been reported for magnetic iron oxide systems where adsorbate–adsorbate interactions and progressive surface coverage lead to a linear decrease in adsorption heat, reflecting the influence of surface heterogeneity and magnetic particle dispersion [243].

### 9.5. Sips Model

The Sips isotherm, also known as the Langmuir-Freundlich model, is a hybrid adsorption isotherm that integrates the empirical heterogeneity of the Freundlich model with the monolayer saturation of the Langmuir model. This combination makes it particularly effective for describing adsorption on heterogeneous surfaces, such as iron oxide nanoparticles, as shown in Table 5 [149].

At low equilibrium concentrations Ce, it approximates the Freundlich behavior, accommodating multilayer adsorption and surface heterogeneity; at high Ce, it transitions to Langmuir-like monolayer saturation, thereby overcoming the Freundlich model’s limitation of a continuously increasing adsorption capacity without a theoretical limit. The nonlinear form is expressed as:

(7) $$q_{\;e}=q_{m,s}\frac{{\left(KₛC_{e}\right)}^{\beta s}}{1+{\left(KₛC_{e}\right)}^{\beta s}}$$

where $q_{m,s}$ is the Sips maximum adsorption capacity, Kₛ is the Sips affinity constant, and *β_s_* is the Sips heterogeneity exponent. The model parameters are sensitive to pH, temperature, and initial concentration, with values often varying between linear and nonlinear regression methods, which enhances its versatility for optimizing nanofiber adsorbent performance.

For example, Magnetic Fe_3_O_4_/ZnO nanocomposites demonstrated good agreement with the Sips model for tetracycline and Congo red adsorption, which is attributed to their porous surfaces and engineered heterogeneity [244].

Collectively, these studies highlight that magnetic nanostructure properties—such as porosity, functionalization, aggregation behavior, and surface heterogeneity—play a significant role in shaping equilibrium adsorption profiles and influencing the selection and performance of isotherm models for emerging organic and inorganic pollutants.

While equilibrium isotherms describe the adsorption capacity and surface energetics at steady state, they do not provide information about the rate at which equilibrium is achieved. Therefore, kinetic modeling is necessary to complement equilibrium analysis and to elucidate the mechanisms controlling contaminant uptake over time.

## 10. Adsorption Kinetics Models

Building upon equilibrium analysis, adsorption kinetics models provide insight into the dynamic aspects of contaminant removal, including rate-limiting steps and mass-transfer mechanisms. The sorption treatment process is governed by various factors, including the adsorbent’s surface properties, solute concentration, and temperature, which collectively influence the overall efficiency of contaminant removal. To comprehensively understand the adsorption mechanism and rate-limiting steps, kinetic models provide crucial insights into adsorption mechanisms and rate-limiting steps, enabling a deeper understanding of the underlying physical and chemical interactions [245].

Magnetic nanostructures can measurably influence adsorption kinetics because their surface chemistry and transport behavior determine which step controls the overall uptake rate. In many MNM systems, abundant functional groups and accessible active sites promote stronger adsorbate–surface interactions, which are frequently reflected in a superior fit to the pseudo-second-order model and $q_{e}$ values consistent with experimental data, suggesting that surface reaction/chemisorption contributions can dominate under certain conditions [240].

However, MNMs may also follow pseudo-first-order kinetics when uptake is governed largely by physisorption and external mass transfer, highlighting that the “best” kinetic model depends on the specific surface functionalization and pollutant speciation [241].

Intraparticle diffusion analysis further shows that adsorption onto magnetic nanocomposites can proceed through multiple sequential steps (boundary-layer/film diffusion followed by diffusion into pores/active sites), evidenced by multilinear $q_{t}$ vs. $t^{1/2}$ plots that do not pass through the origin, indicating that pore diffusion is not the sole rate-limiting mechanism [240].

Finally, operational features often used with MNMs (e.g., intensified mixing or ultrasound-assisted contact) can accelerate the approach to equilibrium by enhancing mass transfer and increasing effective adsorbate–adsorbent interactions, thereby increasing apparent kinetic constants and shortening equilibrium times.

Together, the integration of equilibrium and kinetic modeling provides a comprehensive framework for understanding adsorption performance in MNMs, enabling rational material design and optimization.

### 10.1. Pseudo-First-Order (PFO) Model

This expression, also known as Lagergren’s model, is widely used to describe the adsorption kinetics of sorbates from aqueous solutions onto solid adsorbents, particularly in nanofiber-based systems for contaminant removal. It assumes that the rate-determining step is the diffusion of the adsorbate from the solution to the external surface of the adsorbent, with the adsorption rate proportional to the number of available unoccupied sites. The PFO model effectively fits experimental data for the initial stages of adsorption, providing insights into the rate-limiting mechanisms:

(8) $$q_{t}=q_{e}(1-e^{\;-k_{1}t})$$

where $q_{e}\;$ and $q_{t}\;$ are the amounts of contaminant adsorbed onto the solid matrix at equilibrium and time t (mg∙g^−1^), respectively, and k_1_ (min^−1^) is the PFO rate constant.

### 10.2. Pseudo-Second-Order (PSO) Model

The PSO model is widely used to describe chemisorption processes, assuming that the adsorption rate is proportional to the square of the number of unoccupied sites on the adsorbent surface. The PSO model provides valuable insights into the rate-limiting mechanisms, particularly in nanofiber-based systems for contaminant removal. It often yields a better fit than the PFO model for experimental data and is expressed as:

(9) $$q_{t}=\frac{k_{2}q_{e}^{2}t}{1+{k_{2}q}_{e}t}$$

where $k_{2}$ is (g∙mg^−1^ min^−1^) is the PSO rate constant.

### 10.3. Intra-Particle Diffusion Model

This model, proposed by Weber and Morris, describes the diffusion of the sorbate from the aqueous phase into the pores of the solid adsorbent matrix, providing insights into whether pore diffusion is the rate-limiting step. It is particularly useful for heterogeneous adsorbents, where multistep diffusion processes occur:

(10) $$q_{t}=k_{int}t^{0.5}+C$$

where $k_{int}\;$ (mg∙g^−1^ min^−0.5^) is the intra-particle diffusion rate constant indicative of diffusion efficiency, and C is the intercept representing the boundary layer thickness or external mass transfer effects. A linear plot of $q_{\;t}$ versus $t^{0.5}$ that passes through the origin (C ≈ 0) confirms intra-particle diffusion as the sole rate-limiting mechanism; otherwise, multiple mechanisms (e.g., film diffusion followed by pore diffusion) control the process.

## 11. Future Perspectives

The increasing threat posed by organic and inorganic pollutants entering drinking water supplies has made it imperative to develop sustainable nanotechnology-based remediation approaches to meet the environmental needs of humans, wildlife, and ecosystems. Numerous investigations have examined NMs, which exhibit promising adsorption and photocatalytic performance due to their large surface area, tunable surface functionality, size, shape, and magnetic separability [246]. Among these, MNPs such as zero-valent iron, magnetite, maghemite, and M-type strontium hexaferrite have been well studied.

Despite this progress, the generation of potential secondary pollutants during synthesis, together with the need for extensive toxicity and life-cycle analyses to ascertain long-term impacts on human health, animal welfare, and environmental sustainability, remains a major challenge. Moreover, scalability issues in terms of cost, material stability, process reproducibility, and integration into water treatment systems still hinder large-scale deployment. Regulatory limitations also pose a significant obstacle as standardized regulations governing the environmental release, recovery, and disposal of MNMs remain limited, thereby restricting their translation from the laboratory to real-world applications across various regional or national jurisdictions.

**Figure 3:**
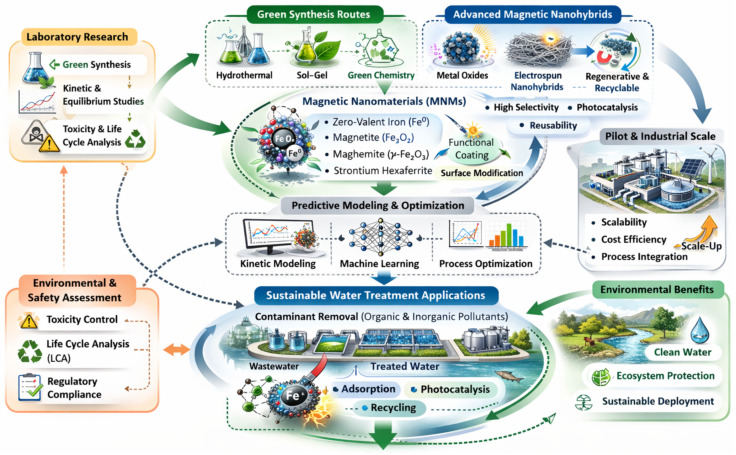
Conceptual framework of future perspectives for MNMs in sustainable water treatment.

Future research should therefore focus on bridging laboratory-scale kinetic and equilibrium optimization with pilot- and industrial-scale implementation (see Figure 3) by emphasizing the design of green synthesis routes, the development of multifunctional magnetic nanohybrid with higher selectivity and regenerative properties, and predictive kinetic modeling to optimize adsorption and photocatalytic systems. Recent advances in electrospun nanohybrids and magnetically recyclable nanophotocatalysts demonstrate promising pathways toward scalable and reusable wastewater treatment technologies [246,247,248], supporting the transition from laboratory-scale investigations to practical environmental applications.

## 12. Conclusions

Magnetic nanomaterials demonstrate significant potential for the removal of organic and inorganic contaminants due to their high surface area, tunable chemistry, structural versatility, and magnetic separability. Their adsorption and photocatalytic performance are closely linked to their nanostructure design, which governs surface interactions, kinetic behavior, and regeneration capability.

Kinetic modeling using the PFO, PSO, and intra-particle diffusion models provides critical insights into rate-limiting steps and adsorption mechanisms, supporting the optimization of material performance. Although advances in nanocomposite fabrication and nanofiber synthesis have improved reactivity and recoverability, challenges related to scalability, cost, long-term stability, and environmental safety remain.

Overall, MNMs represent a promising platform for sustainable water remediation, provided that future research prioritizes green synthesis, enhanced durability, and practical system integration to enable large-scale deployment.

## Figures and Tables

**Table 1:** Merits and demerits of different nanoparticle synthesis methods.

Method	Merits	Demerits	Ref.
Ball milling	Low cost; widely used; scalable process	Requires elevated temperatures; potential contamination from milling media	[58]
Laser evaporation	Chemical-free process; high-purity products; good stoichiometric control	High equipment cost; limited scalability; complex instrumentation	[59]
Wire explosion	Environmentally friendly; low energy consumption; high product purity	Produces polydisperse nanoparticles; limited control over size distribution	[60]
Electron beam lithography	Enables fabrication of nanoscale patterns and two-dimensional geometries with high precision	Requires complex instrumentation; limited throughput; high operational cost	[61]
Coprecipitation	Simple and reproducible; produces ultrafine powders without intermediate phases; cost-effective	Requires strict pH control; possible residual impurities if insufficiently washed	[62]
Sol–gel	Versatile; low-temperature processing; high crystallinity and purity	Limited production yield; relatively long processing time	[63]
Spray pyrolysis	Compatible with various precursors; high purity; good control of particle size distribution	Requires high processing temperatures; possible minor product contamination	[64]
Self-combustion	Simple and rapid; produces single-phase powders; energy-efficient	Limited control over particle size; high local reaction temperatures	[65]
Thermal decomposition	Excellent control over particle size and morphology; high crystallinity	Requires elevated reaction temperatures; high energy consumption; organic solvent use	[66]
Microemulsion	Thermodynamically stable system; uniform particle dispersion; narrow size distribution	Limited production yield; requires surfactants and organic solvents	[67]
Hydrothermal	Simple process; high crystallinity; good control of morphology	Requires elevated temperature and pressure conditions; specialized autoclave equipment	[68]
Solvothermal	Minimizes adsorption of anions and cations; reduces contamination; good size control	Requires elevated temperatures; organic solvent handling	[69]
Pechini	Low toxicity precursors; fine powder formation; good compositional control; high purity	Long processing time; limited material yield; possible minor contamination	[70]
Polyol	Precise control over nucleation and growth; nanometric particle size; low agglomeration	Slow reaction kinetics; limited scalability.	[71]
Self-assembly	Enables fabrication of sub-20 nm molecular patterns; atomically precise nanostructures	Difficult design and fabrication; limited large-scale applicability	[72]
Laser pyrolysis	Produces very small particles; rapid synthesis	Poor crystallinity due to short residence time; broad size distribution	[73]
Microwave	Energy-efficient; controllable reaction parameters; short processing time	Possible variation in physicochemical properties; limited scalability	[74]
Arc discharge	Cost-effective; catalyst-free process	Limited control over particle size and shell thickness; not suitable for industrial scale	[75]
Sonochemical	Rapid synthesis; ultrafine powder formation	Possible structural defects; impurity incorporation; limited uniformity	[76]
Oxidation reduction	Fast reaction rates; simple chemistry	Gas contamination risk; high capital and operational costs	[77]
Chemical vapor deposition	Controllable deposition rate; suitable for industrial-scale production; high surface-to-volume ratio	Requires complex equipment; possible structural defects	[78]
Combustion synthesis	Simple and rapid; energy-efficient; scalable	Generates gaseous emissions (CO_2_, CO); limited size control	[79]

**Table 2:** Removal of different organic contaminants using MNPs and their different adsorption capacities. Most efficiencies were obtained under laboratory conditions; performance may decrease in real wastewaters due to competing solutes and Natural Organic Matter (NOM). The isotherm models are Freundlich (F), Langmuir (L), Dubinin-Radushkevich (D-R), Temkin (T), and Sips (S).

Adsorbent Material	Synthesis Method	Removal Mechanism	Pollutant(s)	Isotherm Model	Removal Efficiency (%, mg/g)	Ref.
TiO_2_/Fe_3_O_4_	-	Adsorption	Sulfamethazine	L; F	88.00%	[83]
0.67BiFeO_3_–0.33BaTiO_3_	Sol–Gel	Catalysis	Methylene Blue, Rhodamine and Crystal violet.	L	Mb: 98.00% Rb: 91.00% Cv: 88.00%	[84]
Graphene-magnetite functionalized diatomite	-	Adsorption	Organochlorine-pesticides	L	97.00%	[85]
CoFe_2_O_4/_PMS	Hydrothermal and Coprecipitation	Catalysis	Phenol sulfonic acid	-	90.00%	[86]
Fly-AsFe_3_O_4_	Precipitation	Adsorption	Red Dye	L; F	153 mg/g	[87]
NiFe_2_O_4_	Coprecipitation	Photocatalysis	Titan Yellow	L	98.80%	[88]
COFe_2_O_4_/SnO_2_	Sol–Gel	Photocatalysis	Indigo carmine dye	-	85%	[89]
Fe3O_4_/HMIL	-	Adsorption	Coomassie brilliant blue R-250	L; F	~93.00–98.00%	[90]
Magnetic MPANI@La	Oxidation-polymerization	Adsorption	Phosphate ions	L	92.49%	[91]
NiO/Co@C_magnetic_	Solvothermal	Adsorption	Organic nitrogen pesticides	L	62.20 mg/g	[92]
CAF@Fe_3_O_4_	Green	Photocatalysis	Methyl orange	L	100.00%	[93]
Fe/Zr-MOFs	Solvothermal	Adsorption	Doxycycline hydrochloride	F	87.50%	[94]
CoFe_2_O_4_@ZnMOF/Graphene	Precipitation	Photocatalysis	Diazinón	L	97.38%	[95]
Fe_3_O_4_@IL	Coprecipitation	Adsorption	Ionic silver	F	100.00%	[96]
Fe_3_O_4_@MgO	Microwave	Photocatalysis	Rhodamine B	L	99.00%	[97]
Zr-doped Fe_3_O_4_	Solvothermal	Photocatalysis	Diazinon Phosphorus	-	97.50%	[98]
a-Fe_2_O_3_/Cu_2_O	Hydrothermal	Photocatalysis	Benzotriazoles	-	100.00%	[99]
SBH-Fe_3_O_4_	Co-precipitation	Adsorption	Chloroquine	L	98.84 mg/g	[100]
Fe_3_O_4_/CA	Green/coprecipitation	Photocatalyst	Metylene Blue	-	93.14%	[101]
NdFeO_3_	Sol–gel-citrate	Photacatalyst	Lomefloxacin and methylene blue	L; F	Lf: 88.00% Mb: 95%	[102]
BiOI-Fe_3_O_4_	Coprecipitation	Photocatalytic	Polystyrene	-	73.00%	[103]
AHA-Fe_3_O_4_	Hydrothermal	Chemisorption	Tetracycline	L; F	91.36%	[104]
Fe-doped TiO_2_@Fe_3_O_4_	Sol–Gel	Photocatalysis	Metronidazole	-	99.37%	[105]
TiO_2_–Fe_3_O_4_	Hydrothermal and Microwave	Photocatalysis	Metronidazole	-	90.00%	[106]
Fe_3_O_4_@TiO_2_-P25	Oxidative precipitation	Photocatalytic	Metoprolol	L	87.00%	[107]
HC-FeNPs	Microwave-assisted	Adsorption	Ethoprophos Terbufos and Diazinon (DIA)	L	95.00%	[108]
CTS@Fe_3_O_4_	Coprecipitation	Adsorption	Caffeic acid Gallic acid Melanoidin	L	Ca: 185 mg/g Ga: 160 mg/g Mel: 580 mg/g	[109]
Nd and Mn co-doped SrFe_12_O_19_	Microemulsion	Photocatalysis	Crystal violet	-	90.70%	[110]
Fe_3_O_4_@Phe	Green	Adsorption	Ciprofloxacin	L	49.27 mg/g	[111]
Co_0.5_Cu_0.5_Fe_2_O_4_	Coprecipitation	Photocatalisys	Tetracycline	-	86.00%	[112]
Fe_3_O_4_@SiO_2_@Salg	-	Adsorption	Organo phosphorus	L	80.50–100.00%	[113]
CoFe_2_O_4_/WS2/PMS	Coprecipitation	Catalyst	Sulfathiazole	L	97.12%	[114]
Mn_2_O_3_ and Co_3_O_4_	Thermal decomposition	Catalysts	Xylene	-	90.00%	[115]
BC/Fe_3_O_4_	Coprecipitation	Photocatalyst	Dye mixture	-	92.19%	[116]
BC/Fe_3_O_4_	Coprecipitation	Adsorption	Methyl orange(MO)	L; F	83.50%	[117]
α-Fe_2_O_3_/TiO_2_	Sonochemical	Photocatalyst	Tetracycline	-	97.50%	[118]
TiO_2_ doped Fe	Sol–Gel	Photodegradation	Congo red dye	-	99.00%	[119]
CoFe_2_O_4_@MOF-5)	Coprecitation	Photodegradation	Metronidazole and penicillin-G	L; F	MTZ: 91.71% PCG: 89.31%	[120]
SnFe_2_O_4_@ZIF-8	Precipitation	Photodegradation	sulfamethoxazole, ciprofloxacin, ampicillin, erythromycin	L	90.00%	[121]
Fe_3_O_4_	Hydrothermal	Photodegradation	Methylene Blue	L; F	90.00%	[122]
Fe_3_O_4_-HKUST-1	Hydrothermal	Photodegradation	Azo	L; F	44.65 mg/g	[123]
Base cross-linked magnetic resin CH-EP@Fe_3_O_4_/AC	Coprecipitation	Adsorption	Malachite green (MG), Reactive red 120 (RR120),	F	MG: 146.30 mg/g RR120: 140.70 mg/g	[124]
Cit-Fe_3_O and @TiO_2_	Coprecipitation and solvothermal	Photocatalysis	Eosin-Methylene Blue	L	Eosin: 96.00% Blue: 82.00%	[125]
Fe_2_O_4_@AC	Coprecipitation	Photocatalysis	Methylene Blue Congo Red	-	99.90%	[126]
VFe_2_O_4_@g-C_3_N_4_	Coprecipitation	Photodegradation	Sulfamethoxazole, Chloramphenicol Ciprofloxacin	-	SUF: 100.00% CIP: 94.00% CIF: 90.00%	[127]
Prosopis juliflora, impregnated with magnetic nanoparticles	Coprecipitation	Photodegradation	Congo red (CR)	F	98.55%	[128]
Fe_3_O_4_	Coprecipitation	Photodegradation	Anionic azo	L	99.99%	[129]
Si@Fe	Green Method	Biodegradation	Malachite Green and polyethylene	L	Vm: 98.11 Poly: 82.92%	[130]
Mg_0.5_Co_0.5_Fe_2_O_4_	Green Method	Photodegradation	Congo Red	L	93.00%	[131]
Zn_0.5_Ni_0.5_FeCrO_4_	Sol–gel/Green	Photodegradation	4-nitrophenol and aniline	L	Nit: 80.00% Anil: 95.00%	[132]
NiFe_2_O_4_	Green Method	Photodegradation	Methylene blue (MB); Azo	L; F	97.00%	[133]
PANI/GO/MOF-Fe_3_O_4_	-	Photodegradation	MO and Naproxen Sodium (NAP)	L; F	239.78 mg/g 40.64 mg/g	[134]
α-Fe_2_O_3_@MgO	Hydrothermal	Photocatalysis	Crystal violet (CV)	L	99.00%	[135]
MoS_2_ NPs Fe_3_O_4_/Cs/MoS_2_/Lac NPs	Hydrothermal	Photocatalysis	Laccase	F	96.80%	[136]
CMC/Ge/citrate@Fe_3_O_4_	Ionotropic Gelation	Adsorption/photocatalysis	Ciprofloxacin	L	96.00%	[137]
MIL-101(Fe)@NiFe_2_O_4_	Hydrothermal	Photocatalysis	Levofloxacin	F	90.00%	[138]
CoFe_2_O_4_	Solvothermal	Photocatalysis	Glycolysis	L	100.00%	[139]
Fe_2_O_3_/TiO_2_	Sol–Gel	Photocatalysis	Methylene Blue	L	MB: 97.71%	[140]
TiO_2_/Fe_2_O_3_	Sol–Gel	Photocatalysis	Methyl Orange	-	Mo: 94.00%	[141]
g-C_3_N_4_@Fe_3_O_4_ and BNNS@Fe_3_O_4_	Coprecipitation	Adsorption	Polyethylene, polystyrene	-	Polyet: 93.70% Polys: 86.56%	[142]
Fe_3_O_4_-Ce@BC/PS	Hydrothermal Coprecipitation	Photocatalysis	Soil Polycyclic aromatic hydrocarbons (PAHs)	-	89.34%	[143]
SrFe_12_O_19_-Fe_3_O_4_	Polymeric precursor	Photocatalysis	Remazol Red ultra Red/Green/Blue (RGB) dye	L	100.00%	[144]
Ag@CoFe_2_O_4_/ h-BN	Hydrothermal and Microwave-assisted	Photocatalysis	Nitrophenols	-	90.00%	[145]
ZnFe_2_O_4_	Green hydrothermal	Photocatalyst	Tetracycline	-	94.00%	[146]
rGO/AK/Fe_3_O_4_	Coprecipitation and ultrasonication	Photocatalyst	Methylene blue	L, F, T, & D-R	98.20%	[147]
LP-CDs@Fe_3_O_4_	Hydrothermal	Photocatalyst	Methylene blue (MB)	-	98.00%	[148]
Corn cobs (CC), iron oxide (IO)	Coprecipitation	Photocatalyst	Triclosan	L; S	94.20%	[149]
ZnFe_2_O_4_/ZnO	Combustion	Photocatalyst	Congo Red	-	90.00%	[150]
ZnFe_2_O_4_/TiO_2_ p-n	Sol–gel	Photocatalyst	Ammonia nitrogen	-	98.52%	[151]

**Table 3:** Removal of different inorganic contaminants using MNPs and their different adsorption capacities. Most efficiencies were obtained under laboratory conditions; competing ions and complex matrices can reduce removal performance. The isotherm models are Freundlich (F), Langmuir (L), Dubinin-Radushkevich (D-R), Temkin (T) and Redlich-Peterson (R-P).

Adsorbent Material	Synthesis Method	Adsorption Mechanism	Pollutant(s)	Isotherm Model	Removal Efficiency (%, mg/g)	Ref.
GO/Fe–Mn	Precipitation	Adsorption	Lead (II)	L	99.00%	[153]
Fe_3_O_4_-Bentonite	Coprecipitation	Adsorption	Chrome (VI)	L	96.50%	[154]
Inulin-Fe_3_O_4_	Ultrasonic	Adsorption	Co^2+^ Cu^2+^ Hg^2+^	L	152.5 mg/g 167.7 mg/g 19.8 mg/g	[155]
Fe@NSC	Coprecipitation	Adsorption	Arsenic (III) As(V)	L	129.54 mg/g 178.65 mg/g	[156]
mPAC-SH magnetic	Hydrothermal	Adsorption	Mercury (II)	L	99.44%	[157]
Fe_3_O_4_-OP-CS	Hydrothermal carbonization	Adsorption	Copper (II) Lead (II)	L	Cu:92.40% Pb: 94.10%	[158]
γ-Fe_2_O_3_ and Fe_3_O_4_	Coprecipitation	Adsorption	Chromium and Cupper	L	92.00%	[159]
Ni Fe_2_O_4_-TiO_2_	Pechini	Photocatalyc	Arsenic (III)	L	97.50%	[160]
Fe_3_O_4_-OP-CS	Chemical Precipitation	Adsorption	Cadmium (II)	L	92.00%	[161]
Fe_3_O_4_@TpPa-NO_2_	-	Adsorption	Lead (II)	L	909.1 mg/g	[162]
Fe_3_O_4_	Thermal -descomposition	Adsorption	Lead (II), Nickel(II), and Cadmium(II)	L	27.18 mg/g	[163]
Fe_3_O_4_NPs and Fe/CuNPs AgNPs	Coprecipitation	adsorption	Lead (II), Copper(II), Cadmium (II) and Nickel (II)	D-R, F, L, and T	Pb: 98.39% Cu: 75.52% Cd: 51.54% Ni: 45.34%	[164]
Fe–Mn/GO	Hydrothermal	Oxidation and Absorption	As (III), As (V)	-	90.00~97.00%	[165]
Fe/Zr	Hydrothermal	Adsorption	As(III) and As(V)	L	AS (III) 99.00% As(V) 99.80%	[166]
Si-Fe-GO	Sol–Gel	Adsorption	Uranium	L	90.20%	[167]
Fe_3_O_4_@PSBC	Pyrolysis	Absorption	Chrome (VI)	L	209 mg/g	[168]
(SiO_2_) with Fe_3_O_4_	Sol–gel	Adsorption	Chrome (VI)	L	64.80%	[169]
Co_3_O_4_	Co-precipitation	Adsorption	Lead (II)	L	99.44%	[170]
MnFe_2_O_4_@SBA-15-(CH_2_)_3_	Hydrothermal	Adsorption	As(V), Cd(II), and Lead (II)	R-P	96.00%	[171]
MoS_2_/Fe_3_O_4_	Solvothermal	Adsorption	Mercury (II)	L; F	97.00%	[172]
Cu Fe_2_O_4_	Pechini-Sol–gel	Adsorption	Cu (II)	L	377.36 mg/g	[173]
CS-Fe_3_O_4_/GO	Coprecipitation	Adsorption	Niquel (II)	L	81.21%	[174]
Fe_3_O_4_-ACH	Coprecipitation	Adsorption	Cr(VI)	L	94.10%	[175]
Fe_3_O_4_@NR-TMD-G1, Fe_3_O_4_@NR-TMD-G2	Co-precipitation	Adsorption	Lead (II) and Cadmium(II)	F, L and D-R	98.50%, 93.60%	[176]
NTs/PEI@alginate@NiFe_2_O_4_	Hydrothermal	Adsorption	Zn^2+^and Pb^2+^	L	Zn: 74.70% Pb: 97.09%	[177]
Fe_3_O_4_/ZnO	Pyrolisis	Adsorption	Chrome(VI) and Lead (II)	L	66.23% 384.62 mg/g	[178]
ZIF-7 and MnFe_2_O_4_	Precipitation and hydrothermal	Adsorption	Co^2+^	L	99.05%	[179]
Fe_3_O_4_/Mg-Al LDOs/AlS (SMA)	Coprecipitation	Adsorption	Chrome (VI) Cu (II)	L	235.3 mg/g 669.2 mg/g	[180]
ZnO@Fe_3_O_4_	Hydrothermal	Adsorption	Lead (II) and Cadmium (II)	-	Lead (II): 99.20–100.00% Cad: 99.60–100.00%	[181]
Fe_3_O_4_@AHA MNPs	Chemical Coprecipitation hydrothermal	Adsorption	La (III)	L	90.00%	[182]
(mGO/CS) and mGO/PA	Coprecipitation	Adsorption	Cr(VI) Pb(II)	L	95.00%	[183]
Cr_0_._5_CoFe_1_._5_O_4_	Hydrothermal	Adsorption	La(III) Ce(III) Sm(III) Eu(III)	L	La: 11.51 mg/g Ce: 11.51 mg/g Sm: 14.62 mg/g Eu: 14.62 mg/g	[184]
BC/Al(OH)3-Fe_3_O_4_-NC	-	Adsorption	Co(II), Cd(II), Sr(II)	L	Co: 99.45%, Cd: 99.65% Sr: 99.78%	[185]
AC@Fe_3_O_4_	Coprecipitation	Adsorption	Cd^2+^, Cu^2+^, Pb^2+^ As^3+^	L; F	Pb: 89.70% Cu: 83.80% Cd: 82.80% As: 80.90%	[186]
Fe_3_O_4_@MCLS	Coprecipitation	Adsorption	Cr(VI)	L	90.00%	[187]

**Table 4:** Advantages and disadvantages of methods for obtaining nanofibers (1D).

Method	Advantages	Disadvantages	Ref.
Electrospinning	Fibers with nanometric sizes, low-cost technology, high surface ratio, high porosity, and improved mechanical properties.	Jet instability, limited control of pore size	[195]
Self-Assembly	It is a direct method for making multifunctional nanofibers.	Complex process, high cost, low productivity.	[196]
Phase Separation	Well-defined pore architecture and size with low equipment demands	Restricted to certain polymer systems, unsuitable for the fabrication of long continuous fibers	[197]
Template Synthesis	Templates of different sizes are used to create fibers of different diameters.	Problem in removing the pavilion	[198]
Drawing	Minimum equipment requirement	Nanofibers smaller than 100 nm cannot be obtained	[199]
Centrifugal Spinning	Versatile, low-cost, and high-production-rate process.	Difficulty in collecting the manufactured material	[200]

**Table 5:** Removal of organic and inorganic contaminants using polymeric nanofibers.

Adsorbent Material	NP Synthesis Method	Nanofiber Synthesis Method	Pollutant(s)	Adsorption Mechanism	Isotherm Model	Capacity Adsorption (%, mg/g)	Ref.
			**Contaminants**	**Organic**			
Bi_2_WO_6_/BiOBr/PAN	Solvothermal	Electrospinning	Levofloxacin	Photocatalysis	L	95.25%	[201]
Magnetic PVA-CNF	Coprecipitation	Electrospinning	Methyl Orange	Adsorption	-	60.00%	[202]
Fe-doped TiO_2_	Solvothermal	Electrsopinning	Methyl Blue	Adsorption and Photocatalysis	-	94.00%	[203]
BiFeO_3_@CdS	Hydrothermal	Electrsopinning	bisphenol A	Catalysis	L	99.70%	[204]
IOC (Act-IOC)Fe_3_O_4_	Pyrolysis	Electrsopinning	Tetracycline and ciprofloxacin	Catalysis	L	Tc: 92.00% Cip: 95.00%	[205]
CoFe_2_O_4_/BiOCl	Solvothermal	Electrsopinning	Rhodamine B, Norfloxacin.	Photocatalysis	L	RhB: 92.90% Nor:75.50%	[206]
α-Fe_2_O_3_/PAN/CaCO_3_/CTA (FPCC)	-	Electrospinning	Methylene Blue, Methyl Orange	Photocatalysis	L	Mb: 96.00% Mo: 95.00%	[207]
WO2.72@Fe_3_O_4_@cellulose	Solvothermal	Free-drying	Methyl Orange	Photocatalysis	-	85.00%	[208]
FeCuOx/eggshell	Coprecipitation	Electrospinning	Carbamazepine	Catalysis	-	85.90%	[209]
Fe doped TiO_2_	Sol–gel	Electrospinning	Rhodamine B, methylene blue, Congo red and methyl orange	Photocatalysis	L	97.00% 99.00%	[210]
Polyacrylonitrile@carbon/MIL-101(Fe)	Hydrothermal	Electrsopinning	Tetracycline	Adsorption	F	392.64 mg/g	[211]
PDA-IL NFsM	-	Electrospinning	Mycotoxins	Adsorption	L	83.00%	[212]
Fe_3_O_4_@GA/PVC	Coprecipitación	Phase separation	Reactive Red-195, Reactive Blue (RB19), and Rifampicin (Rif) antibiotic	Adsorption	L	RR195: 98.30% Rif: 96.50% RB19: 95.60%	[213]
Cel/α-Fe_2_O_3_-ZnO	Hydrothermal	Electrospinning	Reactive black 5 Rb5	Adsorption	L	Rb5: 99.30 mg/g	[214]
g-C_3_N_4_/PAN/PANI@LaFeO_3_	-	Electrospinning	Methylene blue, Methyl violet, Ciprofloxacin and Acetamiprid, Escherichia coli, Staphylococcus aureus	Adsorption	L	MB: 97.00% MV: 94.30% CIP: 87.60% AP: 88.90% *E. coli* 100.00%, S.aur: 80.00%	[215]
MnxFe_2_-xO_4_	Sonochemical	Electrospinning	Methylene Blue	Catalysis	L	>80.00%	[216]
SiO_2_@Fe_3_O_4_@ PS.	-	Electrospinning	Methylene blue	Photocatalytic	-	99.90%	[217]
FeCo-CoFe_2_O_4_	Sol–gel	Electrospinning	Norfloxacin	Photocatalytic	-	93.8%	[218]
ZnO/NiFe_2_O_4_/BiOBr 3D	Solvothermal	Electrospinning	Rhodamine B	Photocatalytic	L	99.61%	[219]
SiO_2_/Ti_3_C_2_ MXene/Fe_3_O_4_	Sol–gel	Electrospinning	Doxorubicin and Meyltne Blue	Photocatalytic degradation	-	>90.00%	[220]
PCL-PEI-Fe_3_O_4_	Hydrothermal	Electrospinning	Congo-Red	Adsorption	L	397.43 mg/g	[221]
Sn doped α-Fe_2_O_3_	-	Electrospinning	Ciprofloxacin and Methylene blue	Photodegradation	L	Cip: 79.80% Mb: 82.70%	[222]
PA6/PANI/α-Fe_2_O_3-x_	Ultrasonication & hydrothermal	Electrospinning	Tetracycline	Photodegradation	-	94.89%	[223]
			**Contaminants**	**Inorganic**			
La_2_O_3_–CeO_2_–Fe_3_O_4_	-	Template-Electrospinning	Fluoride	Adsorption	L	229.89 mg/g	[224]
Fe_3_O_4_@NC@MnO_2_	Hydrothermal	Electrospinning	Re (VII) As (V)	Adsorption	L	10.9 mg/g 236.3 mg/g	[225]
MnFe-LDH/MnFe_2_O_3_@_3_DNF	Hydrothermal	Electrospinning	Cr(VI), Pb(II) and As(III)	Adsorption	L and F	Cr: 79.00% Pb: 84.00% As: 73.00%	[226]
GO/CMC/FeNPs	Hydrothermal	Electrospiining	Pb	Adsorption	D-R	1850 mg/g	[227]
ONPs in a PVA	-	Electrospinning	As(V)	Adsorption	L	80.00%	[228]
PAN/gCN-NH_2_/Fe_3_O_4_: PCNFe	Solvothermal	Electrospinning	As(III) and As(V)	Adsorption	L	As(III): 97.00% As(V): 99.00%	[229]
AOP/ZIF-90@TA/ZVI (AZ@TZ)	-	Electrospinning	Uranium	Catalysis	L	140.06 mg/g	[230]
PB/SiO_2_-NH_2_ NFs	-	Electrospnning	Cs^+^	Adsorption	F	75.36%	[231]
Poly-Fe modified GCE	-	Electrospinning	Cr (VI)	electrochemically	-	90.00%	[232]
Bi_2_WO_6_ nanosheet/CuFe_2_O_4_	Hydrothermal	Electrospinning	Cr (VI) to Cr (III)	Photocatalysis	-	90.30- 96.04%	[233]
KC/TiO_2_-Fe_3_O_4_	Precipitation	Nanoparticles	As (III)	Adsorption	L	92.00–90.00%	[234]
AF-Fe_3_O_4_ NPs	-	Electrospinning	Cr (VI)	Adsorption	F	212.1 mg/g	[235]
PAN/Fe_3_O_4_@CTAB	-	Electrospinning	As (V)	Adsorption	L	97.00%	[236]
Hematite/poly(ε-caprolactone) and chitosan	Coprecipitation	Electrospinning	Cr^6+^ and Cs^+^	Adsorption	L and F	Cr^6+^: 89.84% Cs^+^: 94.71%	[237]
PET-Fe_3_O_4_	Coprecipitation	Electrospinning	Cu^2+^	Adsorption	-	19.8 m/mg	[238]

## Data Availability

No new data were created or analyzed in this study. Data sharing is not applicable to this article.

## Abbreviations

The following abbreviations are used in this manuscript:

MNMs	Magnetic nanomaterials
NMs	Nanomaterials
MNPs	Magnetic nanoparticles
NPs	Nanoparticles
PFOA	Perfluorooctanoic Acid
(e^−^-h^+^)	electron–hole pairs
NOM	Natural Organic Matter
F	Freundlich model
L	Langmuir model
D-R	Dubinin-Radushkevich model
T	Temkin model
S	Sips model
R-P	Redlich-Peterson
$q_{e}$	Amount of pollutant adsorbed per unit mass of adsorbent at equilibrium
$C_{e}$	Equilibrium concentration of the pollutant in solution
PFO	Pseudo-First-Order Model
PSO	Pseudo-Second-Order

## References

[1] Israilova E., Voronina A., Shatila K. (2023). Impact of water scarcity on socio-economic development. E3S Web Conf..

[2] Vallinayagam S., Rajendran K., Lakkaboyana S.K., Remya S.K., Sharma V.K., Kumar V., Venkateswarlu K., Koduru J.R. (2021). Recent developments in magnetic nanoparticles and nano-composites for wastewater treatment. J. Environ. Chem. Eng..

[3] Khan I., Saeed K., Khan I. (2019). Nanoparticles: Properties, applications and toxicities. Arab. J. Chem..

[4] Sung H., Rudowicz C. (2003). Physics behind the magnetic hysteresis loop—A survey of misconceptions in magnetism literature. J. Magn. Magn. Mater..

[5] Martínez-Pedrero F. (2020). Static and dynamic behavior of magnetic particles at fluid interfaces. Adv. Colloid Interface Sci..

[6] Kotnala R.K., Shah J. (2015). Ferrite Materials. Handbook of Magnetic Materials.

[7] Cullity B.D., Graham C.D. (2007). Introduction to Magnetic Materials.

[8] Yamato M., Kimura T. (2020). Magnetic Processing of Diamagnetic Materials. Polymers.

[9] Amorim C.O. (2025). A Compendium of Magnetic Nanoparticle Essentials: A Comprehensive Guide for Beginners and Experts. Pharmaceutics.

[10] Zhang Y., Ortiz J., He S., Li X., Kaur B., Cao B., Seiden Z., Wu S., Wei H. (2025). Magnetically Retrievable Nanoparticles with Tailored Surface Ligands for Investigating the Interaction and Removal of Water-Soluble PFASs in Natural Water Matrices. Sensors.

[11] Reddy L.H., Arias J.L., Nicolas J., Couvreur P. (2012). Magnetic nanoparticles: Design and characterization, toxicity and biocompatibility, pharmaceutical and biomedical applications. Chem. Rev..

[12] Patankar K.K., Jadhav P., Gayakvad K. (2022). Introduction and applications of magnetic nanoparticles. Fundamentals and Industrial Applications of Magnetic Nanoparticles.

[13] Velásquez A., Urquijo J. (2021). Synthesis and characterization of magnetite-maghemite nanoparticles in presence of polyethylene glycol obtained by mechanical milling. Mater. Sci. Eng. B.

[14] Bedoya P.A.C., Botta P.M., Bercoff P.G., Fanovich M.A. (2023). Influence of the milling materials on the mechanochemical synthesis of magnetic iron oxide nanoparticles. J. Alloys Compd..

[15] Zhang D., Gökce B. (2017). Perspective of laser-prototyping nanoparticle-polymer composites. Appl. Surf. Sci..

[16] Piotto V., Litti L., Omelyanchik A., Martucci A., Riello P., Peddis D., Meneghetti M. (2022). Synthesis of magnetic nanoparticles by laser ablation of strontium ferrite under water and their characterization by optically detected magnetophoresis supported by BEM calculations. J. Mater. Chem. C Mater. Opt. Electron. Devices.

[17] Kotov Y.A. (2003). Electric Explosion of Wires as a Method for Preparation of Nanopowders. J. Nanoparticle Res..

[18] Song K., Kim W., Suh C.-Y., Shin D., Ko K.-S., Ha K. (2013). Magnetic iron oxide nanoparticles prepared by electrical wire explosion for arsenic removal. Powder Technol..

[19] Tseng A.A., Chen K., Chen C.D., Ma K.J. (2003). Electron beam lithography in nanoscale fabrication: Recent development. IEEE Trans. Electron. Packag. Manuf..

[20] Jia C.-J., Sun L.-D., Luo F., Han X.-D., Heyderman L.J., Yan Z.-G., Yan C.-H., Zheng K., Zhang Z., Takano M. (2008). Large-Scale Synthesis of Single-Crystalline Iron Oxide Magnetic Nanorings. J. Am. Chem. Soc..

[21] Tan P.T., Hien L.T.T., Anh N.N., Minh P.N., Van Trinh P., Van Hao N. (2025). Graphene oxide–carbon nanotube-magnetite nanocomposites for efficient arsenic removal from aqueous solutions. RSC Adv..

[22] Sajjad A., Hussain S., Jaffari G.H., Hanif S., Qureshi M.N., Zia M. (2023). Fabrication of Hematite (α-Fe_2_O_3_) nanoparticles under different spectral lights transforms physio chemical, biological, and nanozymatic properties. Nano Trends.

[23] Parashar M., Shukla V.K., Singh R. (2020). Metal oxides nanoparticles via sol–gel method: A review on synthesis, characterization and applications. J. Mater. Sci. Mater. Electron..

[24] Tahir M., Fakhar-E-Alam M., Atif M., Mustafa G., Ali Z. (2023). Investigation of optical, electrical and magnetic properties of hematite α-Fe_2_O_3_ nanoparticles via sol-gel and co-precipitation method. J. King Saud Univ. Sci..

[25] Rabenau A. (1985). The Role of Hydrothermal Synthesis in Preparative Chemistry. Angew. Chem. Int. Ed. Engl..

[26] da Silva A.O., Campos A.F.C., Rodrigues M.O., Sousa M.H. (2023). Tuning magnetic and luminescent properties of iron oxide@C nanoparticles from hydrothermal synthesis: Influence of precursor reagents. Surf. Interfaces.

[27] Li C., Wei Y., Liivat A., Zhu Y., Zhu J. (2013). Microwave-solvothermal synthesis of Fe_3_O_4_ magnetic nanoparticles. Mater. Lett..

[28] Sahadevan J., Sojiya R., Padmanathan N., Kulathuraan K., Shalini M., Sivaprakash P., Muthu S.E. (2022). Magnetic property of Fe_2_O_3_ and Fe_3_O_4_ nanoparticle prepared by solvothermal process. Mater. Today Proc..

[29] Mosayebi J., Kiyasatfar M., Laurent S. (2017). Synthesis, functionalization, and design of magnetic nanoparticles for theranostic applications. Adv. Health Mater..

[30] Bozkurt G. (2020). Synthesis and Characterization of α-Fe_2_O_3_ nanoparticles by microemulsion method. Erzincan Üniversitesi Fen Bilim. Enstitüsü Derg..

[31] Koventhan C., Kumar N.K.R., Chen S.-M., Pandi K., Sangili A. (2021). Polyol mediated synthesis of hexagonal manganese cobaltate nanoparticles for voltammetric determination of thioridazine. Colloids Surf. A Physicochem. Eng. Asp..

[32] Anit J., Praveena M., Thoufeeq S., Al-Omari I., Anantharaman M. (2023). A simple polyol one-shot synthesis of Maghemite and Hematite from inexpensive precursors. Inorg. Chem. Commun..

[33] Sun S., Zeng H. (2002). Size-Controlled Synthesis of Magnetite Nanoparticles. J. Am. Chem. Soc..

[34] Agarwal P., Preethi J., Bora D.K. (2021). Direct thermal decomposition of FeCl3.6H2O in oleic acid forms hematite cube and nano octahedron structure with quasicrystalline and supercell symmetries for enhanced photoelectrochemical functionality. Mater. Chem. Phys..

[35] Tahir M.B., Rafique M., Rafique M.S., Nawaz T., Rizwan M., Tanveer M. (2020). Photocatalytic nanomaterials for degradation of organic pollutants and heavy metals. Nanotechnology and Photocatalysis for Environmental Applications.

[36] Bomatí-Miguel O., Mazeina L., Navrotsky A., Veintemillas-Verdaguer S. (2008). Calorimetric study of maghemite nanoparticles synthesized by laser-induced pyrolysis. Chem. Mater..

[37] Pechini M.P. (1963). Method of Preparing Lead and Alkaline Earth Titanates and Niobates and Coating Method Using the Same to Form a Capacitor. U.S. Patent.

[38] Murillo-Ortíz R., Mirabal-García M., Cruz-Rivera J., Valdez-Pérez D., Martínez J., Pérez-Moreno F., Lobo-Guerrero A. (2019). Properties and arsenic removal evaluation of polyvinyl alcohol nanofibers with embedded strontium hexaferrite nanoparticles. Mater. Chem. Phys..

[39] Ateia E.E., Elsayed K., Ramadan R. (2022). Tuning the properties of Ba-M hexaferrite BaFe11.5Co0.5O19: A road towards diverse applications. J. Inorg. Organomet. Polym. Mater..

[40] Wang L., Gao L. (2010). Self-assembly behavior of hematite nanoparticles with controllable anisotropic morphology. J. Colloid Interface Sci..

[41] Bomati O., Morales M., Serna C., Veintemillas S. (2003). Magnetic nanoparticles prepared by laser-induced pyrolysis. IEEE International Digest of Technical Papers on Magnetics Conference.

[42] Criveanu A., Dumitrache F., Fleaca C., Gavrila-Florescu L., Lungu I., Morjan I.P., Socoliuc V., Prodan G. (2023). Chitosan-coated iron oxide nanoparticles obtained by laser pyrolysis. Appl. Surf. Sci. Adv..

[43] Khan S., Sayed M., Sohail M., Shah L.A., Raja M.A., Satinder A. (2019). Chapter 6—Advanced Oxidation and Reduction Processes. Advances in Water Purification Techniques.

[44] Ozkaya T., Baykal A., Koseoğlu Y., Kavas H. (2009). Synthesis of Co_3_O_4_ nanoparticles by oxidation-reduction method and its magnetic characterization. Open Chem..

[45] Desai P.P., Radha M., Savitha G., Boregowda R. (2024). Versatile strategies for multifaceted nanoparticle synthesis—An overview. Nanotechnology and In Silico Tools.

[46] Nashaat A.M., Abu El-Fadl A., Kassem M.A., Nakamura H. (2023). Optimizing a microwave-combustion synthesis and particle-size dependent magnetic properties of M-type Sr ferrite. Mater. Chem. Phys..

[47] Fernández-Pacheco R., Arruebo M., Marquina C., Ibarra R., Arbiol J., Santamaría J. (2006). Highly magnetic silica-coated iron nanoparticles prepared by the arc-discharge method. Nanotechnology.

[48] Karami H., Goli F., Ordoukhanian J. (2016). Synthesis of magnetite/hematite/iron nanocomposites by the low voltage arc discharge in water in the presence of external magnetic field. Int. J. Electrochem. Sci..

[49] Suslick K. (2003). Sonochemistry. Comprehensive Coordination Chemistry II.

[50] Yadav V.K., Ali D., Khan S.H., Gnanamoorthy G., Choudhary N., Yadav K.K., Thai V.N., Hussain S.A., Manhrdas S. (2020). Synthesis and characterization of amorphous iron oxide nanoparticles by the sonochemical method and their application for the remediation of heavy metals from wastewater. Nanomaterials.

[51] Aruna S.T., Mukasyan A.S. (2008). Combustion synthesis and nanomaterials. Curr. Opin. Solid State Mater. Sci..

[52] Căpraru A., Moacă E.-A., Păcurariu C., Ianoş R., Lazău R., Barbu-Tudoran L. (2021). Development and characterization of magnetic iron oxide nanoparticles using microwave for the combustion reaction ignition, as possible candidates for biomedical applications. Powder Technol..

[53] Bube R. (2001). Cadmium Sulfide and Telluride. Encyclopedia of Materials: Science and Technology.

[54] Levish A., Joshi S., Winterer M. (2023). Chemical vapor synthesis of nanocrystalline iron oxides. Appl. Energy Combust. Sci..

[55] Li Z., Xu S., Xiao G., Qian L., Song Y. (2019). Removal of hexavalent chromium from groundwater using sodium alginate dispersed nano zero-valent iron. J. Environ. Manag..

[56] Hübner U., Spahr S., Lutze H., Wieland A., Rüting S., Gernjak W., Wenk J. (2024). Advanced oxidation processes for water and wastewater treatment—Guidance for systematic future research. Heliyon.

[57] Sandhu S., Zumeit A., Tian Z., Vinciguerra V., Dahiya R. (2025). Semiconductor manufacturing wastewater challenges and the potential solutions via printed electronics. iScience.

[58] Fecht H.J., Hellstern E., Fu Z., Johnson W.L. (1990). Nanocrystalline metals prepared by high-energy ball milling. Met. Trans. A.

[59] Amendola V., Barcikowski S. (2017). A quarter-century of nanoparticle generation by lasers in liquids: Where are we now, and what’s next?. J. Colloid Interface Sci..

[60] Kurlyandskaya G., Madinabeitia I., Beketov I., Medvedev A., Larrañaga A., Safronov A., Bhagat S. (2014). Structure, magnetic and microwave properties of FeNi nanoparticles obtained by electric explosion of wire. J. Alloys Compd..

[61] Chen Y.F. (2015). Nanofabrication by electron beam lithography and its applications: A review. Microelectron. Eng..

[62] Marciello M., Luengo Y., Morales M.P. (2016). Iron oxide nanoparticles for cancer diagnosis and therapy. Nanoarchitectonics for Smart Delivery and Drug Targeting.

[63] Khan I., Morishita S., Higashinaka R., Matsuda T.D., Aoki Y., Kuzmann E., Homonnay Z., Katalin S., Pavić L., Kubuki S. (2021). Synthesis, characterization and magnetic properties of ε-Fe_2_O_3_ nanoparticles prepared by sol-gel method. J. Magn. Magn. Mater..

[64] Meierhofer F., Mädler L., Fritsching U. (2020). Nanoparticle evolution in flame spray pyrolysis—Process design via experimental and computational analysis. AIChE J..

[65] Cubas P.d.J., Semkiw A.W., Monteiro F.C., Weinert P.L., Monteiro J.F.H.L., Fujiwara S.T. (2020). Synthesis of CuCr_2_O_4_ by self-combustion method and photocatalytic activity in the degradation of Azo Dye with visible light. J. Photochem. Photobiol. A Chem..

[66] Glasgow W., Fellows B., Qi B., Darroudi T., Kitchens C., Ye L., Crawford T.M., Mefford O.T. (2016). Continuous synthesis of iron oxide (Fe_3_O_4_) nanoparticles via thermal decomposition. Particuology.

[67] Shang Z., Xu P., Feng T., Sun Y., He K., Li G., Li X. (2024). Probe into a novel surfactant-free microemulsion system of ethylene glycol monobutyl ether + water + diesel for crude oil removal and recovery from oily sludge. Sci. Total Environ..

[68] Tazim T.Q., Kawsar M., Hossain M.S., Bahadur N.M., Ahmed S. (2025). Hydrothermal synthesis of nano-metal oxides for structural modification: A review. Next Nanotechnol..

[69] Wei J., Gao Z., Song Y., Yang W., Wang J., Li Z., Mann T., Zhang M., Liu L. (2013). Solvothermal synthesis of Li–Al layered double hydroxides and their electrochemical performance. Mater. Chem. Phys..

[70] Loghman-Estarki M., Torkian S., Rastabi R.A., Ghasemi A. (2017). Effect of annealing temperature and copper mole ratio on the morphology, structure and magnetic properties of Mg0.5−xCuxZn0.5Fe_2_O_4_ nanoparticles prepared by the modified Pechini method. J. Magn. Magn. Mater..

[71] Fievet F., Lagier J., Figlarz M. (1989). Preparing monodisperse metal powders in micrometer and submicrometer sizes by the polyol process. MRS Bull..

[72] Borah R., Ag K.R., Minja A.C., Verbruggen S.W. (2023). A review on self-assembly of colloidal nanoparticles into clusters, patterns, and films: Emerging synthesis techniques and applications. Small Methods.

[73] Lungu I.I., Andronescu E., Dumitrache F., Gavrila-Florescu L., Banici A.M., Morjan I., Criveanu A., Prodan G. (2023). Laser pyrolysis of iron oxide nanoparticles and the influence of laser power. Molecules.

[74] Saxena V., Chandra U., Chandra U. (2011). Microwave synthesis: A physical concept. Microwave Heating.

[75] Retnosari I., Hayati I.N., Amalia A., Hastuti S., Saraswati T.E. (2018). The chemical characteristics of iron oxide/carbon synthesized by the arc discharge method in liquid media with the addition of ammonia. J. Kim. Sains Dan Apl..

[76] Khan M.M. (2025). Sonochemical synthesis method. Photocatalysts: Synthesis and Characterization Methods.

[77] Iida H., Nakanishi T., Takada H., Osaka T. (2006). Preparation of Magnetic Iron-Oxide Nanoparticles by Successive Reduction–Oxidation in Reverse Micelles: Effects of Reducing Agent and Atmosphere. Electrochim. Acta.

[78] Besmann T., Stinton D., Lowden R. (1988). Chemical vapor deposition techniques. MRS Bull..

[79] Kumar A., Cross A., Manukyan K., Bhosale R., Broeke L.v.D., Miller J., Mukasyan A., Wolf E. (2015). Combustion synthesis of copper–nickel catalysts for hydrogen production from ethanol. Chem. Eng. J..

[80] Li X., Shen X., Jiang W., Xi Y., Li S. (2024). Comprehensive review of emerging contaminants: Detection technologies, environmental impact, and management strategies. Ecotoxicol. Environ. Saf..

[81] Mukhopadhyay A., Duttagupta S., Mukherjee A. (2022). Emerging organic contaminants in global community drinking water sources and supply: A review of occurrence, processes and remediation. J. Environ. Chem. Eng..

[82] Sharma M., Kalita P., Senapati K.K., Garg A. (2018). Study on magnetic materials for removal of water pollutants. Emerging Pollutants—Some Strategies for the Quality Preservation of Our Environment.

[83] Al-Salihi S., Bayati M., Jasim A.M., Fidalgo M.M., Xing Y. (2022). Magnetic mesoporous TiO_2_/Fe_3_O_4_ nanocomposite adsorbent for removal of sulfamethazine from water. Environ. Adv..

[84] Muduli S.P., Veeralingam S., Badhulika S. (2022). Free-standing, non-toxic and reusable 0.67BiFeO3–0.33BaTiO_3_ based polymeric piezo-catalyst for organic dye wastewater treatment. J. Water Process. Eng..

[85] Sanad M.M., Gaber S.E., El-Aswar E.I., Farahat M.M. (2023). Graphene-magnetite functionalized diatomite for efficient removal of organochlorine pesticides from aquatic environment. J. Environ. Manag..

[86] Shi B., Wang Y., Ahmed I., Zhang B. (2022). Catalytic degradation of refractory phenol sulfonic acid by facile, calcination-free cobalt ferrite nanoparticles. J. Environ. Chem. Eng..

[87] Harja M., Lupu N., Chiriac H., Herea D.-D., Buema G. (2022). Studies on the removal of congo red dye by an adsorbent based on Fly-Ash@Fe_3_O_4_ mixture. Magnetochemistry.

[88] Bameri I., Saffari J., Baniyaghoob S., Ekrami-Kakhki M.-S. (2022). Synthesis of magnetic nano-NiFe_2_O_4_ with the assistance of ultrasound and its application for photocatalytic degradation of Titan Yellow: Kinetic and isotherm studies. Colloid Interface Sci. Commun..

[89] AbouSeada N., Ahmed M., Elmahgary M.G. (2022). Synthesis and characterization of novel magnetic nanoparticles for photocatalytic degradation of indigo carmine dye. Mater. Sci. Energy Technol..

[90] Ezzat A.O., Tawfeek A.M., Rajabathar J.R., Al-Lohedan H.A. (2022). Synthesis of new hybrid structured magnetite crosslinked poly ionic liquid for efficient removal of Coomassie Brilliant Blue R-250 dye in aqueous medium. Molecules.

[91] Rezania S., Kadi A., Kamyab H., Ghfar A.A., Nodeh H.R., Ibrahim W.N.W. (2022). Lanthanum doped magnetic polyaniline for removal of phosphate ions from water. Chemosphere.

[92] Zhao J., Huang P., Wang X., Yang J., Zhou Z., Du X., Lu X. (2022). Efficient adsorption removal of organic nitrogen pesticides: Insight into a new hollow NiO/Co@C magnetic nanocomposites derived from metal-organic framework. Sep. Purif. Technol..

[93] Manojkumar M., Jeyajothi K., Jagadeesan A., Jeevanantham V. (2022). Magnetic separation of green synthesized Fe_3_O_4_ nanoparticles on photocatalytic activity of methyl orange dye removal. J. Indian Chem. Soc..

[94] Wei F., Wang K., Li W., Ren Q., Qin L., Yu M., Liang Z., Nie M., Wang S. (2023). Preparation of Fe/Ni-MOFs for the adsorption of ciprofloxacin from wastewater. Molecules.

[95] Roostaee M., Sheikhshoaie I. (2022). Synthesis of CoFe2O4 @ZnMOF/Graphene Nanoflake for Photocatalytic Degradation of Diazinon Under Visible Light Irradiation: Optimization and Modeling Using a Fractional Factorial Method.

[96] Muñoz-Sandoval M.J., Caravaca M., López-García I., Hernández-Córdoba M., Vicente-Martínez Y. (2022). Complete and simultaneous removal of ionic silver and silver nanoparticles by using an ionic liquid supported on a magnetic nanoparticle core. Environ. Res..

[97] Rayaroth M.P., Oh D., Lee C.-S., Chang Y.-S. (2022). Simultaneous removal of heavy metals and dyes in water using a MgO-coated Fe_3_O_4_ nanocomposite: Role of micro-mixing effect induced by bubble generation. Chemosphere.

[98] Zheng W., Sun Y., Gu Y. (2022). Catalysis and adsorption of Zr-doped Fe_3_O_4_ nanoparticles provide a new strategy for diazinon removal and phosphorus recovery from aqueous solution. J. Environ. Chem. Eng..

[99] Han J.-H., Jia W.-H., Liu Y., Wang W.-D., Zhang L.-K., Li Y.-M., Sun P., Fan J., Hu S.-T. (2022). α-Fe_2_O_3_/Cu_2_O composites as catalysts for photoelectrocatalytic degradation of benzotriazoles. Water Sci. Eng..

[100] Vidovix T.B., Januário E.F.D., Bergamasco R., Vieira A.M.S. (2022). Evaluation of agro-industrial residue functionalized with iron oxide magnetic nanoparticles for chloroquine removal from contaminated water. Mater. Lett..

[101] Bassim S., Mageed A.K., AbdulRazak A.A., Majdi H.S. (2022). Green synthesis of Fe_3_O_4_ nanoparticles and its applications in wastewater treatment. Inorganics.

[102] Shankara A.H., Prabagar J.S., Tenzin T., Yadav S., Kumar K.M.A., Shivaraju H.P. (2023). Facile synthesis of NdFeO_3_ perovskite for photocatalytic degradation of organic dye and antibiotic. Mater. Today: Proc..

[103] Khairudin K., Abu Bakar N.F., Osman M.S. (2022). Magnetically recyclable flake-like BiOI-Fe_3_O_4_ microswimmers for fast and efficient degradation of microplastics. J. Environ. Chem. Eng..

[104] Zhang J., Li X., Xu H., Zhang W., Feng X., Yao Y., Ma Y., Su L., Ren S., Li S. (2023). Coating magnetic nanoparticles with artificial humic acid derived from rice straw for effective removal of tetracycline antibiotics. Ind. Crop. Prod..

[105] Heidarinejad F., Kamani H., Khtibi A. (2023). Magnetic Fe-doped TiO_2_@Fe_3_O_4_ for metronidazole degradation in aqueous solutions: Characteristics and efficacy assessment. Heliyon.

[106] Kubiak A., Kubacka M., Gabała E., Dobrowolska A., Synoradzki K., Siwińska-Ciesielczyk K., Czaczyk K., Jesionowski T. (2020). Hydrothermally assisted fabrication of TiO_2_-Fe_3_O_4_ composite materials and their antibacterial activity. Materials.

[107] López J., Rey A., Viñuelas-Zahinos E., Álvarez P.M. (2023). Preparation of a new green magnetic Fe_3_O_4_ @TiO_2_-P25 photocatalyst for solar advanced oxidation processes in water. J. Environ. Chem. Eng..

[108] Nassar A.E., El-Aswar E.I., Rizk S.A., Gaber S.E.-S., Jahin H.S. (2023). Microwave-assisted hydrothermal preparation of magnetic hydrochar for the removal of organophosphorus insecticides from aqueous solutions. Sep. Purif. Technol..

[109] Yin J., Fang K., Li J., Du N., Hu D., Cao D., Tian R., Deng L., Li K. (2023). Competitive adsorption mechanisms of pigments in sugarcane juice on starch-based magnetic nanocomposites. Int. J. Biol. Macromol..

[110] Bibi F., Iqbal S., Kalsoom A., Jamshaid M., Ahmed A., Mirza M., Qureshi W.A. (2023). Magnetically separable Nd and Mn co-doped SrFe_12_O_19_ hexa-ferrites nanostructures for the evaluation of structural, magnetic and photo-catalytic studies under solar irradiation for the crystal violet dye removal from the industrial wastes. Ceram. Int..

[111] Fu X., Sarker S., Ma W., Zhao W., Rong Y., Liu Q. (2023). Novel phenylalanine-modified magnetic ferroferric oxide nanoparticles for ciprofloxacin removal from aqueous solution. J. Colloid Interface Sci..

[112] Chen J., Rasool R.T., Ashraf G.A., Guo H. (2023). The stimulation of peroxymonosulfate via novel Co_0.5_Cu_0.5_Fe_2_O_4_ heterogeneous photocatalyst in aqueous solution for organic contaminants removal. Mater. Sci. Semicond. Process..

[113] Liu Z., Zhao H., Wang J., Wang Z., Di S., Xu H., Wang Q., Wang X., Qi P. (2023). Green synthesis of Fe_3_O_4_@SiO_2_@Salg particles for organophosphorus pesticides removal: Mechanisms, biosafety, and application. Chemosphere.

[114] Li Y., Wang Q., Zhang X., Dong L., Yuan Y., Peng C., Zhang M., Rao P., Pervez N., Gao N. (2023). CoFe_2_O_4_/WS_2_ as a highly active heterogeneous catalyst for the efficient degradation of sulfathiazole by activation of peroxymonosulfate. J. Water Process. Eng..

[115] Xia Y., Guo M., Zhao Y., Ren Z., Deng J. (2023). Preparation of Mn_2_O_3_-Co_3_O_4_ and its xylene removal by oxidation. Chem. Phys. Impact.

[116] Santana R.M.d.R., Napoleão D.C., Rodriguez-Diaz J.M., Gomes R.K.d.M., Silva M.G., de Lima V.M.E., Neto A.A.d.M., Vinhas G.M., Duarte M.M.M.B. (2023). Efficient microbial cellulose/Fe_3_O_4_ nanocomposite for photocatalytic degradation by advanced oxidation process of textile dyes. Chemosphere.

[117] Saleh A.K., Salama A., Badawy A.S., Diab M.A., El-Gendi H. (2023). Paper sludge saccharification for batch and fed-batch production of bacterial cellulose decorated with magnetite for dye decolorization by experimental design. Cellulose.

[118] Mohammadi M., Sabbaghi S., Binazadeh M., Ghaedi S., Rajabi H. (2023). Type-1 α-Fe_2_O_3_/TiO2 photocatalytic degradation of tetracycline from wastewater using CCD-based RSM optimization. Chemosphere.

[119] Wahyuni E.T., Lestari N.D., Cinjana I.R., Annur S., Natsir T.A., Mudasir M. (2023). Doping TiO_2_ with Fe from iron rusty waste for enhancing its activity under visible light in the Congo red dye photodegradation. J. Eng. Appl. Sci..

[120] Adeleke B.K., Olalekan O.A., Adewuyi A., Lau W.J., Adeyemi O.G. (2023). Purification of metronidazole and penicillin-G contaminated water by MOF-5 imprinted cobalt ferrite. Results Chem..

[121] Adewuyi A., Ogunkunle O.A., Oderinde R.A. (2023). Synthesis of tin ferrite-dopped zeolitic imidazolate framework and its application in the photocatalytic degradation of sulfamethoxazole, ciprofloxacin, erythromycin and ampicillin in water. Catal. Commun..

[122] Rafie S.F., Sayahi H., Abdollahi H., Abu-Zahra N. (2023). Hydrothermal synthesis of Fe_3_O_4_ nanoparticles at different pHs and its effect on discoloration of methylene blue: Evaluation of alternatives by TOPSIS method. Mater. Today Commun..

[123] Ma Z., Liu C., Srinivasakannan C., Li L., Wang Y. (2023). Synthesis of magnetic Fe_3_O_4_-HKUST-1 nanocomposites for azo dye adsorption. Arab. J. Chem..

[124] Kaveh R., Bagherzadeh M. (2022). Simultaneous removal of mercury ions and cationic and anionic dyes from aqueous solution using epichlorohydrin cross-linked chitosan @ magnetic Fe_3_O_4_/activated carbon nanocomposite as an adsorbent. Diam. Relat. Mater..

[125] Bibi S., Ahmad A., Shah S.S., Siddiq M., Habila M.A., Choi D. (2024). Synthesis of Cit-Fe_3_O_4_@TiO_2_ photocatalyst for the degradation of eosin-Y and methylene blue dye. Ceram. Int..

[126] Chergui F., Mokhtar A., Abdelkrim S., Sardi A., Hachemaoui M., Boukoussa B., Djelad A., Sassi M., Viscusi G., Abboud M. (2024). Optimizing catalytic performance: Reduction of organic dyes using synthesized Fe_3_O_4_@AC magnetic nano-catalyst. Mater. Chem. Phys..

[127] Adewuyi A., Oderinde R.A. (2024). Photocatalytic removal of some selected antibiotics in polluted water system by graphitic carbon nitride-enhanced vanadium ferrite (VFe_2_O_4_@g-C_3_N_4_). Chemosphere.

[128] Deivayanai V., Karishma S., Thamarai P., Saravanan A., Yaashikaa P. (2024). Efficient red azo dye removal from wastewater using magnetic nanoparticle impregnated Prosopis juliflora biomass: ANN modeling approach. Desalination Water Treat..

[129] Ali N.S., Khader E.H., Khudhur R.H., Abdulrahman M.A., Salih I.K., Albayati T.M. (2024). Removal of anionic azo dye from wastewater using Fe_3_O_4_ magnetic nanoparticles adsorbents in a batch system. Desalination Water Treat..

[130] Ariaeenejad S., Barani M., Roostaee M., Lohrasbi-Nejad A., Mohammadi-Nejad G., Salekdeh G.H. (2024). Enhanced pollutant degradation via green-synthesized core-shell mesoporous Si@Fe magnetic nanoparticles immobilized with metagenomic laccase. Int. J. Biol. Macromol..

[131] Harki D.A.H., Naghipour A., Fardood S.T. (2024). Eco-friendly synthesis and characterization of Mg_0.5_Co_0.5_Fe_2_O_4_ magnetic nanoparticles for photocatalytic degradation of congo red dye. Case Stud. Chem. Environ. Eng..

[132] Fardood S.T., Moradnia F., Aminabhavi T.M. (2024). Green synthesis of novel Zn_0.5_Ni_0.5_FeCrO_4_ spinel magnetic nanoparticles: Photodegradation of 4-nitrophenol and aniline under visible light irradiation. Environ. Pollut..

[133] Muhaymin A., Mohamed H.E.A., Hkiri K., Safdar A., Kotsedi L., Maaza M. (2024). Green synthesis of NiFe_2_O_4_ nanoparticles using Hyphaene thebaica: A facile route towards magnetic and photocatalytic application. Mater. Today Chem..

[134] Hussain E., Shahadat M., Ahtesham A., Ibrahim M.N.M. (2024). Synthesis, characterization, and applications of ambi-functional PANI/GO/MOF-Fe_3_O_4_ magnetic nanocomposite for removing industrial dye and emerging contaminant. Sep. Purif. Technol..

[135] Thabet M., El-Monaem E.M.A., Alharbi W.R., Mohamoud M., Abdel-Aty A.-H., Ibrahim I., Abdel-Lateef M.A., Goda A.E., Elnasr T.A.S., Wang R. (2024). Adsorption and photocatalytic degradation activities of a hybrid magnetic mesoporous composite of α-Fe_2_O_3_ nanoparticles embedded with sheets-like MgO. J. Water Process. Eng..

[136] Rezagholizade-Shirvan A., Ghasemi A., Mazaheri Y., Shokri S., Fallahizadeh S., Sani M.A., Mohtashami M., Mahmoudzadeh M., Sarafraz M., Darroudi M. (2024). Removal of aflatoxin M1 in milk using magnetic laccase/MoS_2_/chitosan nanocomposite as an efficient sorbent. Chemosphere.

[137] Ferfera-Harrar H., Sadi A., Benhalima T. (2024). Magnetic recyclable carboxymethyl cellulose/gelatin/citrate@Fe_3_O_4_ photo-nanocomposite beads for ciprofloxacin removal via hybrid adsorption/photocatalysis process under solar light as a renewable energy source. Int. J. Biol. Macromol..

[138] Zou J., Yu Q., Cao D., Wang Q., Ma N., Dai W. (2024). NiFe_2_O_4_ magnetic nanoparticles supported on MIL-101(Fe) as bimetallic adsorbent for boosted capture ability toward levofloxacin. Mater. Today Chem..

[139] Du J.-T., Wu H., Jie Y., Xia Y., Yang Z., Yan H., Wang Q., Wang J.-X., Chen J.-F. (2025). Magnetically recyclable CoFe_2_O_4_ nanocatalysts for efficient glycolysis of polyethylene terephthalate. Chem. Eng. Sci..

[140] Liu L., Cui Z., Feng B., Sui M., Huang H., Wu Z. (2024). Synthesis of Fe_2_O_3_/TiO_2_ photocatalytic composites for methylene blue degradation as a novel strategy for high-value utilisation of iron scales. Materials.

[141] Kartikowati C.W., Syadana D., Ramadikadipura M.M., Puspitasari D.A., Poerwadi B., Fauziyah M., Arutanti O. (2024). Synthesis of TiO_2_/Fe_2_O_3_ nanocomposites as photocatalyst for methyl orange degradation. E3S Web Conf..

[142] Indhur R., Kumar A., Bux F., Kumari S. (2025). Efficient microplastic removal from wastewater using Fe_3_O_4_ functionalized g-C_3_N_4_ and BNNS: A comprehensive study. J. Environ. Chem. Eng..

[143] Deng Z., Wang N., Wang Y., Li X., Li H., Zou D., Liu Y. (2025). Persulfate advanced oxidation based on a novel CeO_2_-Fe_3_O_4_ heterojunction catalyst for enhanced degradation of soil PAHs. Chem. Eng. Sci..

[144] Paulista A.P.F., Barbosa F.F., Júnior M.A.D.N., Cavalcanti W.E.C., Soares J.d.O., Morales M., Pergher S.B., Braga T.P. (2024). Photocatalytic degradation of the remazol red ultra RGB dye using SrFe_12_O_19_-Fe_3_O_4_ magnetic oxides dispersed in silica: Effect of reduction temperature. Desalination Water Treat..

[145] Poormand F., Farhadi S., Zabardasti A., Maleki M., Mahmoudi F. (2025). Silver (Ag) nanoparticles decorated on magnetic CoFe_2_O_4_/h-BN nanocomposites for efficient catalytic removal of toxic nitrophenols. Mater. Today Commun..

[146] Vinayagam R., Batra S., Murugesan G., Goveas L.C., Varadavenkatesan T., Menezes A., Selvaraj R. (2025). Emerging contaminant removal using eco-friendly zinc ferrite nanoparticles: Sunlight-driven degradation of tetracycline. Emerg. Contam..

[147] Ahmed H.M., Kebede W.L., Yaebyo A.B., Digisu A.W., Tadesse S.F. (2025). Adsorptive removal of methylene blue dye by a ternary magnetic rGO/AK/Fe_3_O_4_ nanocomposite: Studying adsorption isotherms, kinetics, and thermodynamics. Chem. Phys. Impact.

[148] Joga S.B., Korabandi D., Lakkaboyana S.K., Kumar V. (2025). Synthesis of iron nanoparticles on lemon peel carbon dots (LP-CDs@ Fe_3_O_4_) applied in Photo-Catalysis, Antioxidant, Antidiabetic, and Hemolytic activity. Inorg. Chem. Commun..

[149] Almeida-Naranjo C.E., Santana-Romo F., Gallegos-Castro E., Villamar-Ayala C.A., Debut A. (2025). Triclosan removal from synthetic solution using corn cobs and their magnetic composites: Insights from batch adsorption and fixed-bed column studies. Ind. Crop. Prod..

[150] Nazeer Z.A.M., Praveen M., Harikrishna R., Kumar M., Nagarajaiah S., Nagabhushana B.M. (2024). Photocatalytic degradation of the azo dye “Congo-Red” by ZnFe_2_O_4_/ZnO nanocomposite. J. Mines Met. Fuels.

[151] Ke S., Naghizadeh M., Sun L., Jin H., Dong S., Huang T. (2025). Highly reactive ZnFe_2_O_4_/TiO_2_ p-n heterojunction photocatalyst accelerates interfacial charge transfer for boosted photodegradation of ammonia nitrogen. Chem. Eng. Sci..

[152] Farrer B.T., Pecoraro V.L. (2003). Bioinorganic Chemistry. Encyclopedia of Physical Science and Technology.

[153] Raj S., Sinha U., Singh H., Bhattacharya J. (2022). Novel GO/Fe–Mn hybrid for the adsorptive removal of Pb(II) ions from aqueous solution and the spent adsorbent disposability in cement mix: Compressive properties and leachability study for circular economy benefits. Environ. Sci. Pollut. Res..

[154] Adisu N., Balakrishnan S., Tibebe H. (2022). Synthesis and characterization of Fe_3_O_4_ -bentonite nanocomposite adsorbent for Cr(VI) removal from water solution. Int. J. Chem. Eng..

[155] Herab A.A., Salari D., Ostadrahimi A., Olad A. (2022). Preparation of magnetic inulin nanocomposite and its application in the removal of methylene blue and heavy metals from aqueous solution. Mater. Chem. Phys..

[156] Kalimuthu P., Kim Y., Subbaiah M.P., Jeon B.-H., Jung J. (2022). Novel magnetic Fe@NSC nanohybrid material for arsenic removal from aqueous media. Chemosphere.

[157] Gheitasi F., Ghammamy S., Zendehdel M., Semiromi F.B. (2022). Removal of mercury (II) from aqueous solution by powdered activated carbon nanoparticles prepared from beer barley husk modified with Thiol/Fe_3_O_4_. J. Mol. Struct..

[158] Hu X., Dai L., Ma Q., Xu J., Ma J., Liu X. (2022). One-pot synthesis of iron oxides decorated bamboo hydrochar for lead and copper flash removal. Ind. Crop. Prod..

[159] Anbalagan K., Kumar M.M., Sudarsan J., Nithiyanantham S. (2022). Removal of Heavy Metal Ions from Industrial Wastewater Using Magnetic Nanoparticles. J. Eng. Res..

[160] Borges G.C.d.C., Verdi I.R., Fidelis M.Z., Junior H.E.Z., Lenzi G.G., de Souza É.C.F., Alves O.C., Brackmann R. (2023). Magnetic heterostructures of NiFe_2_O_4_ and TiO_2_: Pechini synthesis, characterization and photocatalytic performance in arsenite oxidation. J. Water Process. Eng..

[161] Jadhao J.S., Rathod N.V., Rao A., Ghugare C.D., Chavan S.M., Kubade A.V., Kalyani V.S., Patil A.B. (2023). Efficient removal of toxic Cd(II) ions from waste streams by a novel modified biodegradable magnetic sorbent. Chem. Inorg. Mater..

[162] Li Y., Yan X., Yu S., Hou G., Yang J., Bi W., Bie H., Yang C., Mi Q. (2023). Construction of magnetic COF composites for lead removal with fast dynamics and superior capacity. J. Taiwan Inst. Chem. Eng..

[163] Kothavale V., Sharma A., Dhavale R., Chavan V., Shingte S., Selyshchev O., Dongale T., Park H., Zahn D., Salvan G. (2023). Carboxyl and thiol-functionalized magnetic nanoadsorbents for efficient and simultaneous removal of Pb(II), Cd(II), and Ni(II) heavy metal ions from aqueous solutions: Studies of adsorption, kinetics, and isotherms. J. Phys. Chem. Solids.

[164] Şahi̇n M., Atasoy M., Arslan Y., Yildiz D. (2023). Removal of Ni(II), Cu(II), Pb(II), and Cd(II) from aqueous phases by silver nanoparticles and magnetic nanoparticles/nanocomposites. ACS Omega.

[165] Zhang K., Guo F., Graham N., Yu W. (2022). Engineering of 3d graphene oxide hydrogel-supported Mno_2_-feooh nanoparticles with synergistic effect of oxidation and absorption toward highly efficient removal of arsenic. SSRN Electron. J..

[166] Guo Q., Li Y., Zheng L.-W., Wei X.-Y., Xu Y., Shen Y.-W., Zhang K.-G., Yuan C.-G. (2023). Facile fabrication of Fe/Zr binary MOFs for arsenic removal in water: High capacity, fast kinetics and good reusability. J. Environ. Sci..

[167] Huang S., Zhang Y., Mei B., Tian X., Zhu W., Liao J., Sun N. (2023). Construction of mesoporous Si-Fe-GO composite for the highly efficient removal of uranium. Sep. Purif. Technol..

[168] Yu C., Liao Y. (2023). Removal of Cr(VI) ions from wastewater by Fe_3_O_4_-loaded porous sludge biochar. Water Sci. Technol..

[169] Li H., Hua J., Li R., Zhang Y., Jin H., Wang S., Chen G. (2023). Application of magnetic nanocomposites in water treatment: Core–Shell Fe_3_O_4_ material for efficient adsorption of Cr(VI). Water.

[170] Singh S., N P., Naik T., Uppara B., Thamaraiselvan C., Behera S., Kour R., Dwivedi P., Subramanian S., Khan N.A. (2023). Removal of Pb ions using green Co_3_O_4_ nanoparticles: Simulation, modeling, adsorption, and biological studies. Environ. Res..

[171] Loulic F.B., Shirazi R.H.S.M., Miralinaghi M., Panahi H.A., Moniri E. (2023). Highly efficient removal of toxic As(V), Cd (II), and Pb(II) ions from water samples using MnFe_2_O_4_@SBA-15-(CH_2_)_3_-adenine as a recyclable bio-nanoadsorbent. Microporous Mesoporous Mater..

[172] Li S., Yang L., Wu J., Yao L., Han D., Liang Y., Yin Y., Hu L., Shi J., Jiang G. (2023). Efficient and selective removal of Hg(II) from water using recyclable hierarchical MoS_2_/Fe_3_O_4_ nanocomposites. Water Res..

[173] Al-Kadhi N.S., Basha M.T. (2024). Enhanced removal of Cd(II) Ions from aqueous media via adsorption on facilely synthesized copper ferrite nanoparticles. Molecules.

[174] Nguyen T.H., Nguyen V.D., Vu A.-T. (2024). Synthesis of CS-Fe_3_O_4_/GO nanocomposite for adsorption of heavy metal in aqueous environment. Nanotechnology.

[175] Rind I.K., Memon N., Sarı A., Khuhawar M.Y., Tuzen M., Hasan S.N.U., Memon A.A., Soomro W.A., Brohi R.O.Z., Saleh T.A. (2024). Magnetic nanoparticles loaded hydrochar for effective Cr(VI) removal from water: Batch and column studies. Mater. Chem. Phys..

[176] Mirza M., Bodaghifard M.A., Darvish F. (2024). Synthesis of a nitrogen-rich dendrimer grafted on magnetic nanoparticles for efficient removal of Pb(ii) and Cd(ii) ions. RSC Adv..

[177] Motawea E.A., El-Sabban H.A., Kang J.-H., Ko Y.G. (2024). Efficient multifunctional PPy-NTs/PEI@alginate@NiFe_2_O_4_ magnetic beads for heavy metals removal: Experimental design and optimization interpretations. Int. J. Biol. Macromol..

[178] Hinsene H., Bhawawet N., Imyim A. (2024). Rice husk biochar doped with deep eutectic solvent and Fe_3_O_4_/ZnO nanoparticles for heavy metal and diclofenac removal from water. Sep. Purif. Technol..

[179] Yao L., Hong C., Qi Y., Wu L. (2025). Effective Co2+ removal from aqueous solution and industrial wastewater using ZIF-7 and MnFe_2_O_4_-modified walnut shell biochar composite. Sep. Purif. Technol..

[180] Jiang Y., Tian Q., Zhang H., Yue X., Xue S., Qiu F., Zhang T. (2024). One-step removal of anionic/cationic heavy metal ions from wastewater by magnetic amphoteric adsorbent. J. Water Process. Eng..

[181] Korkmaz Ş., Özkan E.H., Uzun D., Yetim N.K., Özcan C. (2025). Magnetic solid phase extraction of lead (II) and cadmium (II) from water samples using ZnO@Fe_3_O_4_ nanoparticles combined with flame atomic absorption spectrometry determination. J. Sep. Sci..

[182] Memon S.A., Duan J., Li W., Wei Y. (2025). Fabrication of core-shell structured Fe_3_O_4_ @amide humic acid magnetic nanoparticles and their highly efficient adsorption for La (III). Sep. Sci. Technol..

[183] Manzoor Q., Farrukh M.A., Qamar M.T., Sajid A., Aldossari S.A., Manikandan A., Iqbal M. (2024). Polymer-assisted synthesis of ternary magnetic graphene oxide nanocomposite for the adsorptive removal of Cr(VI) and Pb(II) ions. Heliyon.

[184] El-Aryan Y. (2025). Facile hydrothermal synthesis and characterization of magnetic Cr-cobalt ferrite nanocomposite as a novel adsorbent for lanthanide ions. Inorg. Chem. Commun..

[185] Arabkhani P., Asfaram A. (2025). A novel biowaste-derived magnetic adsorbent for efficient removal of cadmium, cobalt and strontium ions from industrial wastewater. Inorg. Chem. Commun..

[186] Alhameedawi F.A.H., Maybodi A.S., Khathi M.T. (2025). Synthesis and characterization of AC@Fe_3_O_4_ nanocomposite and its application as an efficient adsorbent for removal of some heavy metal ions from aqueous solutions. Iran. J. Chem. Chem. Eng..

[187] Alyasi H., Wahib S., Tong Y., Gomez T., Mahmoud K.A. (2025). Magnetic MXene chitosan-lignosulfonate composite (Fe_3_O_4_@ MCLS) for the reductive removal of Cr(VI) and other heavy metals from water. J. Hazard. Mater. Adv..

[188] Behroozi A.H., Al-Shaeli M., Vatanpour V. (2023). Fabrication and modification of nanofiltration membranes by solution electrospinning technique: A review of influential factors and applications in water treatment. Desalination.

[189] Rogina A. (2014). Electrospinning process: Versatile preparation method for biodegradable and natural polymers and biocomposite systems applied in tissue engineering and drug delivery. Appl. Surf. Sci..

[190] Xu D., Samways D.S., Dong H. (2017). Fabrication of self-assembling nanofibers with optimal cell uptake and therapeutic delivery efficacy. Bioact. Mater..

[191] Zhao J., Han W., Chen H., Tu M., Zeng R., Shi Y., Cha Z., Zhou C. (2011). Preparation, structure and crystallinity of chitosan nano-fibers by a solid–liquid phase separation technique. Carbohydr. Polym..

[192] Ugo P., Moretto L.M. (2007). Moretto, Template deposition of metals. Handbook of Electrochemistry.

[193] Saleem H., Trabzon L., Kilic A., Zaidi S.J. (2020). Recent advances in nanofibrous membranes: Production and applications in water treatment and desalination. Desalination.

[194] Chen C., Dirican M., Zhang X. (2019). Centrifugal spinning—High rate production of nanofibers. Electrospinning: Nanofabrication and Applications.

[195] Khan W.S., Asmatulu R., Ceylan M., Jabbarnia A. (2013). Recent progress on conventional and non-conventional electrospinning processes. Fibers Polym..

[196] Gao Y., Shi J., Yuan D., Xu B. (2012). Imaging enzyme-triggered self-assembly of small molecules inside live cells. Nat. Commun..

[197] Zhang H., Ji X., Li P., Liu C., Lou J., Wang Z., Wen W., Xiao Y., Zhang M., Zhu X. (2020). Liquid-liquid phase separation in biology: Mechanisms, physiological functions and human diseases. Sci. China Life Sci..

[198] Garg T., Rath G., Goyal A.K. (2015). Biomaterials-based nanofiber scaffold: Targeted and controlled carrier for cell and drug delivery. J. Drug Target..

[199] Xu T.-C., Han D.-H., Zhu Y.-M., Duan G.-G., Liu K.-M., Hou H.-Q. (2021). High strength electrospun single copolyacrylonitrile (coPAN) nanofibers with improved molecular orientation by drawing. Chin. J. Polym. Sci..

[200] Marjuban S.M.H., Rahman M., Duza S.S., Ahmed M.B., Patel D.K., Rahman S., Lozano K. (2023). Recent advances in centrifugal spinning and their applications in tissue engineering. Polymers.

[201] Wang C., He D., Zhao H., Wang C., Wang K. (2022). Study on high efficiency and fast photodegradation of Bi_2_WO_6_/BiOBr/PAN nanofibrous film. J. Mater. Res. Technol..

[202] Sanchez L.M., Espinosa E., Zélis P.M., Martín R.M., Niza J.d.H., Rodríguez A. (2022). Cellulose nanofibers/PVA blend polymeric beads containing in-situ prepared magnetic nanorods as dye pollutants adsorbents. Int. J. Biol. Macromol..

[203] Van-Pham D.-T., Nhi P.T.Y., Long T.V.B., Nguyen C.-N., Nhan L.M., Quyen T.T.B., Tuyen L.T.C., Mai N.T.N., Thien D.V.H. (2022). Electrospun Fe-doped TiO_2_/chitosan/PVA nanofibers: Preparation and study on photocatalytic and adsorption properties. Mater. Lett..

[204] Long J., Ren T., Han J., Li N., Chen D., Xu Q., Li H., Lu J. (2022). Heterostructured BiFeO_3_@CdS nanofibers with enhanced piezoelectric response for efficient piezocatalytic degradation of organic pollutants. Sep. Purif. Technol..

[205] Kim C.-Y., Kim S.H., An H.-R., Park J.-I., Jang Y., Seo J., Kim H., Son B., Jeong Y., Jeong B. (2023). Iron oxide incorporated carbide nanofiber composites for removal of organic pollutants and heavy metals from water. Ceram. Int..

[206] Liu J., Wang H., Chang M.-J., Sun M., He Z.-W., Zhang C.-M., Zhu W.-Y., Chen J.-L., Du H.-L., Peng L.-G. (2022). Magnetically separatable CoFe_2_O_4_/BiOCl: Controllable synthesis, superior photocatalytic performance and mechanism towards decomposing RhB, NOR and Cr(VI) under visible light. Colloids Surf. A Physicochem. Eng. Asp..

[207] Kumar M., Tiwari A., Randhawa J.K. (2022). Electrospun nanofibers of α-hematite/polyacrylonitrile/calcium carbonate/cellulose triacetate as a multifunctional platform in, wastewater treatment and remineralisation. Desalination.

[208] Chaudhuri S., Wu C.-M., Motora K.G. (2023). Highly efficient solar-light-driven self-floatable WO2.72@Fe_3_O_4_ immobilized cellulose nanofiber aerogel/polypropylene Janus membrane for interfacial photocatalysis. J. Photochem. Photobiol. A Chem..

[209] Gao Y., Zhao Z., Song L., Cao D., Zhou S., Gao T., Shang J., Cheng X. (2023). Eggshell supported Cu doped FeOx magnetic nanoparticles as peroxymonosulfate activator for carbamazepine degradation. Chem. Eng. J..

[210] Mahadadalkar M.A., Park N., Yusuf M., Nagappan S., Nallal M., Park K.H. (2023). Electrospun Fe doped TiO_2_ fiber photocatalyst for efficient wastewater treatment. Chemosphere.

[211] Teng Y., Li W., Wang J., Jia S., Zhang H., Yang T., Li X., Li L., Wang C. (2023). A green hydrothermal synthesis of polyacrylonitrile@carbon/MIL-101(Fe) composite nanofiber membrane for efficient selective removal of tetracycline. Sep. Purif. Technol..

[212] Yang H., Dai H., Chen Y., Wan X., Li F., Xu Q. (2023). Efficient and simple simultaneous adsorption removal of multiple mycotoxins from environmental water. Sep. Purif. Technol..

[213] Vatanpour V., Paziresh S., Behroozi A.H., Karimi H., Esmaeili M.S., Parvaz S., Ghazanlou S.I., Maleki A. (2023). Fe_3_O_4_@Gum Arabic modified polyvinyl chloride membranes to improve antifouling performance and separation efficiency of organic pollutants. Chemosphere.

[214] Aboelfetoh E.F., Aboubaraka A.E., Ebeid E.-Z.M. (2023). Synergistic Effect of iron and copper oxides in the removal of organic dyes through thermal induced catalytic degradation process. J. Clust. Sci..

[215] Mao Y., Lin L., Chen Y., Yang M., Zhang L., Dai X., He Q., Jiang Y., Chen H., Liao J. (2023). Preparation of site-specific Z-scheme g-C3N4/PAN/PANI@LaFeO_3_ cable nanofiber membranes by coaxial electrospinning: Enhancing filtration and photocatalysis performance. Chemosphere.

[216] Fuentes-García J., Sanz B., Mallada R., Ibarra M., Goya G. (2023). Magnetic nanofibers for remotely triggered catalytic activity applied to the degradation of organic pollutants. Mater. Des..

[217] Moatmed S.M., Khedr M., El-Dek S., El-Deen A.G. (2024). Multifunctional nanofiber membrane for high removal efficiency of biological/organic contaminations and oil-in-water emulsion under gravity-driven separation. J. Mol. Liq..

[218] Huang Z., Zhang X., Zhu Z., Guo Z., Wang X., Zhu L., Zhang G., Liu B., Xu D. (2024). Enhanced peroxymonosulfate activation by highly magnetic FeCo-CoFe_2_O_4_ biphasic fibers for norfloxacin degradation. Chem. Eng. J..

[219] Bi F., Zhou B., Li R., Du R., Zheng Z., Fu X., Zhao L., Xiao S., Wang L., Dong X. (2025). An efficient, green, and magnetic recycling wastewater treatment technique enabled by ZnO/NiFe_2_O_4_/BiOBr 3D nanofibers photo-fenton process. Mater. Sci. Semicond. Process..

[220] Rong J., Haipeng B., Ijaz M.F., Shahzad K., Mushtaq S., Iqbal Y. (2025). Multifunctional magnetic SiO_2_/Fe_3_O_4_@MXene nanofiber membranes for reusable removal of doxorubicin and organic dyes from industrial wastewater. Inorg. Chem. Commun..

[221] Amini F., Delavari H.H., Ghovvati S., Poursalehi R. (2025). Electrospinning optimization for enhanced hydrophilicity and surface functionality of Poly(ε-caprolactone)/Polyethyleneimine nanofibers with embedded Fe_3_O_4_ nanoparticles. J. Polym. Res..

[222] Lakshmi C.N., Irfan M., Singh N. (2025). Doping effects of Sn in 1-D α-Fe_2_O_3_ electrospun nanofibers for improved photocatalytic degradation of ciprofloxacin: Kinetics, intermediates identification and mechanisms. Inorg. Chem. Commun..

[223] Wang W., Li M., Si J., Wang Q., Qiu S., Xu Y., Liu X., Cui Z. (2025). Preparation and characterization of PA6/PANI/α-Fe_2_O_3_-x composite nanofiber membranes for the removal of tetracycline from wastewater. J. Water Process. Eng..

[224] Jian S., Shi F., Hu R., Liu Y., Chen Y., Jiang W., Yuan X., Hu J., Zhang K., Jiang S. (2022). Electrospun magnetic La_2_O_3_–CeO_2_–Fe_3_O_4_ composite nanofibers for removal of fluoride from aqueous solution. Compos. Commun..

[225] Huang Y., Zhou J., Zhang Y., Yan L., Bao S., Yin Y., Lu J. (2022). Encapsulating hollow Fe_3_O_4_ in intertwined N-doped carbon nanofibers for high-performance supercapacitors and sodium-ion batteries. J. Alloys Compd..

[226] Poudel M.B., Shin M., Kim H.J. (2022). Interface engineering of MIL-88 derived MnFe-LDH and MnFe_2_O_3_ on three-dimensional carbon nanofibers for the efficient adsorption of Cr(VI), Pb(II), and As(III) ions. Sep. Purif. Technol..

[227] Neskoromnaya E.A., Khamizov R.K., Melezhyk A.V., Memetova A.E., Mkrtchan E.S., Babkin A.V. (2022). Adsorption of lead ions (Pb2+) from wastewater using effective nanocomposite GO/CMC/FeNPs: Kinetic, isotherm, and desorption studies. Colloids Surfaces A Physicochem. Eng. Asp..

[228] Torasso N., Vergara-Rubio A., Pereira R., Martinez-Sabando J., Baudrit J.R.V., Cerveny S., Goyanes S. (2022). An in situ approach to entrap ultra-small iron oxide nanoparticles inside hydrophilic electrospun nanofibers with high arsenic adsorption. Chem. Eng. J..

[229] Tripathy M., Hota G. (2020). Maghemite and graphene oxide embedded polyacrylonitrile electrospun nanofiber matrix for remediation of arsenate ions. ACS Appl. Polym. Mater..

[230] Ye D., Gao Q., Li T., Wu X., Wu Y. (2023). Photo-Fenton catalytic anti-fouling membranes for efficient elimination of radionuclides and organic contaminants. Desalination.

[231] Feng S., Gao J., Li S., Cao X., Ni J., Yue X., Zheng W., Li Y., Hu X., Zhang Y. (2024). Amino modified nanofibers anchored to Prussian blue nanoparticles selectively remove Cs+ from water. J. Environ. Sci..

[232] Halder S., Chakraborty C. (2023). Fe(II)-based metallo-supramolecular polymer nanostructures: An efficient electrode material for highly sensitive electrochemical detection and abatement of hexavalent chromium. J. Environ. Chem. Eng..

[233] Musa M.A., Shao H., Xu D., Sun F., Dong X., Azis R.S., Ugya A.Y., Ari H.A. (2023). Enhanced visible light photocatalytic reduction of Cr (VI) by Bi2WO6 nanosheet/CuFe_2_O_4_ nanofiber heterojunctions. J. Photochem. Photobiol..

[234] Rind I.K., Tuzen M., Sarı A., Lanjwani M.F., Memon N., Saleh T.A. (2023). Synthesis of TiO_2_ nanoparticles loaded on magnetite nanoparticles modified kaolinite clay (KC) and their efficiency for As(III) adsorption. Chem. Eng. Res. Des..

[235] Luo Y., Hu Z., Lei X., Wang Y., Guo X. (2023). Fluorescent magnetic chitosan-based hydrogel incorporating Amino-Functionalized Fe_3_O_4_ and cellulose nanofibers modified with carbon dots for adsorption and detection of Cr (VI). Colloids Surf. A Physicochem. Eng. Asp..

[236] Yang F., Zhu F., Shi H., Dong X., Sheng J., Zhou J. (2025). Magnetic nanofibers in heavy metal arsenic(V) pollution control research. Langmuir.

[237] Khalith M., Karuppannan S.B., Ramalingam R., Raiyan D., Vijayalakshmi S., Arunachalam K.D. (2025). Fabrication and characterization of electrospun nanofibers infused with hematite nanoparticles for the remediation of heavy metals from aqueous medium. Nano-Struct. Nano-Objects.

[238] Hassan A., El-Aziz A.A., Elwi M., Klingner A. (2025). Fabrication of Iron oxide nanoparticle loaded recycled PET nanofibers for water purification applications. Discov. Mater..

[239] Al-Fiydh M.N., Najm H.F., Karam F.F., Baqir S.J. (2024). Thermodynamics, kinetic study and equilibrium isotherm analysis of cationic dye adsorption by ternary composite. Results Chem..

[240] Mashile G.P., Dimpe K.M., Nomngongo P.N. (2020). A Biodegradable Magnetic Nanocomposite as a Superabsorbent for the Simultaneous Removal of Selected Fluoroquinolones from Environmental Water Matrices: Isotherm, Kinetics, Thermodynamic Studies and Cost Analysis. Polymers.

[241] Melhi S. (2023). Recyclable Magnetic Nanocomposites For Efficient Removal Of Cadmium Ions From Water: Performance, Mechanism And Isotherm Studies. Environ. Pollut. Bioavailab..

[242] Cui B., Chen Z., Wang F., Zhang Z., Dai Y., Guo D., Liang W., Liu Y. (2022). Facile Synthesis of Magnetic Biochar Derived from Burley Tobacco Stems towards Enhanced Cr(VI) Removal: Performance and Mechanism. Nanomaterials.

[243] Leone V.O., Pereira M.C., Aquino S.F., Oliveira L.C.A., Correa S., Ramalho T.C., Gurgel L.V.A., Silva A.C. (2018). Adsorption of Diclofenac on a Magnetic Adsorbent Based on Maghemite: Experimental and Theoretical Studies. New J. Chem..

[244] Zhao A., Tang Q., Chen Y., Qiu C., Huang X. (2023). Magnetic Adsorbent Fe_3_O_4_/ZnO/LC for the Removal of Tetracycline and Congo Red from Aqueous Solution. Molecules.

[245] Revellame E.D., Fortela D.L., Sharp W., Hernandez R., Zappi M.E. (2020). Adsorption kinetic modeling using pseudo-first order and pseudo-second order rate laws: A review. Clean. Eng. Technol..

[246] Scaffaro R., Maio A., Gammino M. (2024). Electrospun Polymeric Nanohybrids with Outstanding Pollutants Adsorption and Electroactivity for Water Treatment and Sensing Devices. Adv. Compos. Hybrid Mater..

[247] Patil A.G., Vivekanandan A.K., Hemavathi A.B., Suri S., Pande R., Kagale S., Yadav S., Patil G. (2023). Sustainable Wastewater Treatment Using Magnetic Nanohybrids. IWA Publishing eBooks.

[248] Zheng Z., He J., Zhang Z., Kumar A., Khan M., Lung C.W., Lo I.M.C. (2024). Magnetically Recyclable Nanophotocatalysts in Photocatalysis-Involving Processes for Organic Pollutant Removal from Wastewater: Current Status and Perspectives. Environ. Sci. Nano.

